# Molecular mechanism of specific HLA-A mRNA recognition by the RNA-binding-protein hMEX3B to promote tumor immune escape

**DOI:** 10.1038/s42003-024-05845-y

**Published:** 2024-02-07

**Authors:** Kanglong Yang, Guanglin Chen, Fan Yu, Xianyang Fang, Jiahai Zhang, Zhiyong Zhang, Yunyu Shi, Liang Zhang

**Affiliations:** 1 Hefei National Research Center for Cross disciplinary Science, School of Life Sciences, Division of Life Sciences and Medicine, University of Science and Technology of China, Hefei, Anhui PR China; 2 Ministry of Education Key Laboratory for Membraneless Organelles and Cellular Dynamics, University of Science & Technology of China, Hefei, Anhui PR China; 3 Center for Advanced Interdisciplinary Science and Biomedicine of IHM, University of Science & Technology of China, Hefei, Anhui PR China; 4 Department of Physics, University of Science and Technology of China, Hefei, Anhui PR China; 5 Beijing Advanced Innovation Center for Structural Biology, School of Life Sciences, Tsinghua University, Beijing, PR China

**Keywords:** Cancer therapeutic resistance, Molecular modelling

## Abstract

Immunotherapy, including immune checkpoint inhibitors and adoptive cell transfer, has obtained great progress, but their efficiencies vary among patients due to the genetic and epigenetic differences. Human MEX3B (hMEX3B) protein is an RNA-binding protein that contains two KH domains at the N-terminus and a RING domain at its C-terminus, which has the activity of E3 ubiquitin ligase and is essential for RNA degradation. Current evidence suggests that hMEX3B is involved in many important biological processes, including tumor immune evasion and HLA-A regulation, but the sequence of substrate RNA recognized by hMEX3B and the functional molecular mechanisms are unclear. Here, we first screened the optimized hMEX3B binding sequence on the HLA-A mRNA and reported that the two tandem KH domains can bind with their substrate one hundred times more than the individual KH domains. We systematically investigated the binding characteristics between the two KH domains and their RNA substrates by nuclear magnetic resonance (NMR). Based on this information and the small-angle X-ray scattering (SAXS) data, we used molecular dynamics simulations to obtain structural models of KH domains in complex with their corresponding RNAs. By analyzing the models, we noticed that on the KH domains’ variable loops, there were two pairs of threonines and arginines that can disrupt the recognition of the RNA completely, and this influence had also been verified both in vitro and in vivo. Finally, we presented a functional model of the hMEX3B protein, which indicated that hMEX3B regulated the degradation of its substrate mRNAs in many biological processes. Taken together, our research illustrated how the hMEX3B protein played a key role in translation inhibition during the immune response to tumor cells and provided an idea and a lead for the study of the molecular mechanism and function of other MEX3 family proteins.

## Abstract (web summary)

This study reports RNA recognition by hMEX3B via NMR, SAXS and MD simulation methods. The authors also investigated hMEX3B functions both in vitro and in vivo and proposed how hMEX3B promotes tumor immune escape by regulating the HLA-A.

## Introduction

Lots of progress has been achieved in the field of cancer immunotherapy^1^. Immune checkpoints (ICs), including programmed cell death protein-1 (PD-1), programmed cell death ligand-1 (PDL1), and cytotoxic T lymphocyte-associated antigen-4 (CTLA-4), are a class of immune-modulating proteins involved in the negative regulation of the immune system^2–5^. Immune checkpoint blockade (ICB) specific to immune checkpoint molecules has emerged as one of the most promising approaches in cancer immunotherapy. However, these durable responses are only limited to a subset of patients and the mechanism of why some people cannot respond to immunotherapy efficiently is still unknown^5,6^. Understanding the molecular mechanisms of resistance to immunotherapy is critical for finding new strategies to overcome resistance to immunotherapy. T cells are critical in the adaptive immune system that are able to distinguish between healthy and tumor cells. Upon recognition of tumor-specific antigen fragments (peptides), activated T cells will contribute to the immune response^7,8^. The major histocompatibility complex (MHC) molecules, or human leukocyte antigens (HLA) in humans, bind these antigen peptides to present them to T cells and be recognized by the T cell receptors (TCR) on the surface of T cell. This recognition event is the first step that leads to T cell activation preceding the immune response^9,10^.

In 2018, Huang and colleagues used a kinome library screen and identified hMEX3B, an RNA binding protein as an important regulator in melanoma resistance to PD-1 blockade immunotherapy^11^. Low expression of hMEX3B in melanoma cells was strongly associated with response in a cohort of patients with melanoma treated with anti-PD1 checkpoint blockade. hMEX3B overexpression is associated with resistance to PD-1 blockade.

This effect of hMEX3B was dependent on the endogenous expression of HLA-A and could be reversed by overexpression of exogenous HLA-A. The authors demonstrate that the hMEX3B disrupts HLA-A by binding the 3′UTR of its mRNA. This is an important work that illustrated that antigen presentation is identified as a critical pathway in resistance mechanisms to immune checkpoint blockade and hMEX3B plays an important role in this pathway. Even so, there still existed some unclear questions, such as how the hMEX3B binds to 3′UTR of HLA-A mRNA and how it regulates the expression of HLA-A. To answer these questions, we have done structural biology study and function study for hMEX3B and HLA-A mRNA.

**Fig. 1:**
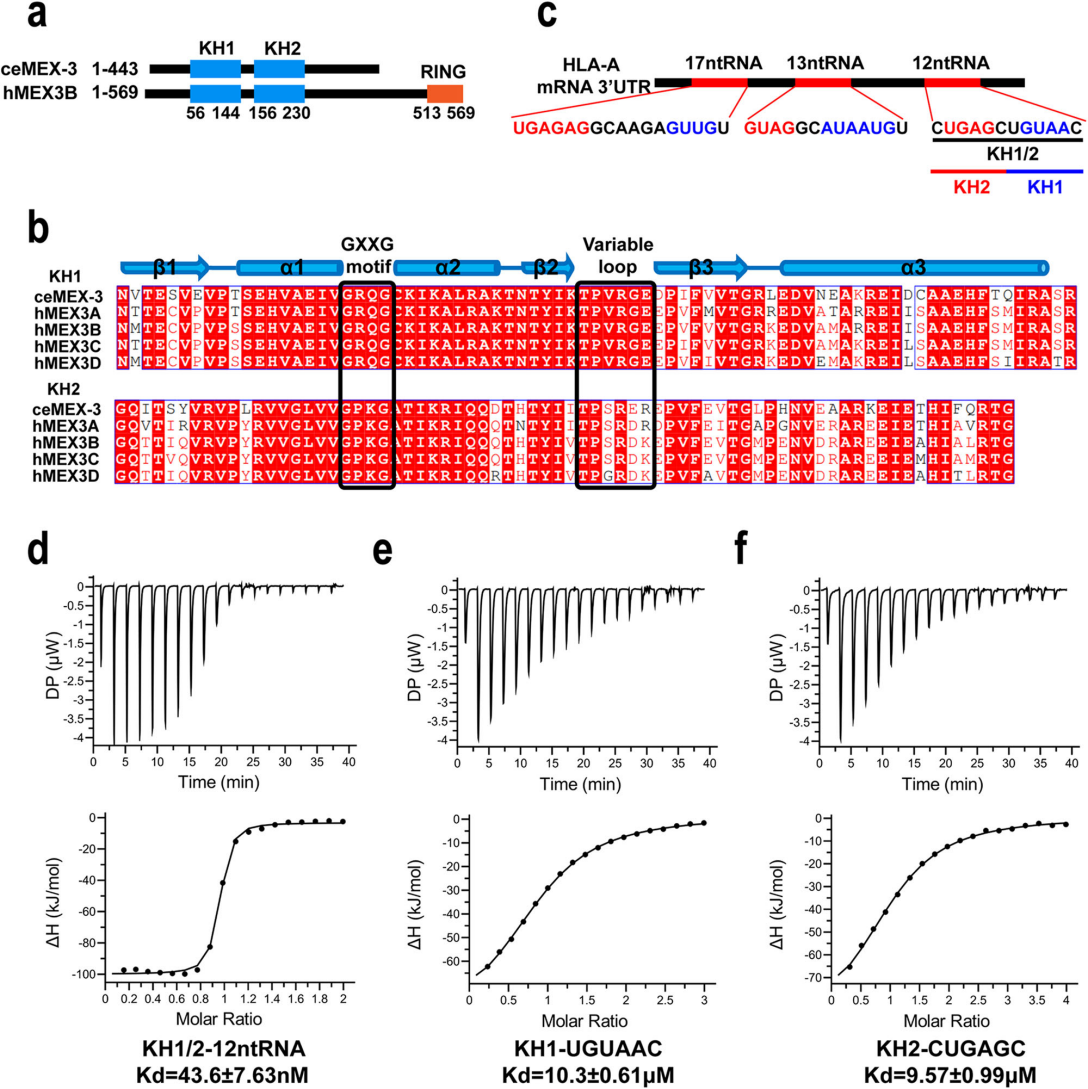
hMEX3B KH domains bind the 12-nt RNA from 3′UTR of HLA-A mRNA. **a** Domain comparison of human hMEX3B protein and *C. elegans* ceMEX-3 protein. The numbers under the KH domains represent the hMEX3B constructs used for this article. **b** Sequence alignment between the two KH domains of hMEX3B and ceMEX-3. Conserved residues are highlighted in red. The GXXG motifs and variable loops are indicated by black squares. The secondary structure elements of the hMEX3B KH domains are shown on the top. **c** The sequences of the three RNA fragments which contained the hMEX3B motifs in the HLA-A mRNA 3′UTR, including the RNA sequence and the location in the full-length HLA-A mRNA 3′UTR. Sequences marked in red represent motif derived RNA nucleotides. The third region represented the 12-nt RNA constructs used in this study. **d** The ITC fitting result of hMEX3B KH1/2 domains with 12-nt RNA. **e**, **f** The ITC fitting results of individual hMEX3B KH1 domain with 6-nt RNA (UGUAAC) and KH2 domain with 6-nt RNA (CUGAGC).

MEX-3 family proteins are RNA-binding proteins (RBPs). In mammals, there are four *mex3* family genes that encode four homologous proteins (hMEX3A ~ hMEX3D). All four hMEX-3 proteins are comprised of N-terminus two tandem KH domains, and the C-terminus, a typical C3HC4-type zinc finger RING domain, that has E3 ubiquitin ligase activity (Fig. 1a). The KH domain is RNA binding domain, exist in both eukaryotes and prokaryotes, contain three α-helices, three β-sheets and a conserved GXXG motif^12–16^. Two KH domains recognize single-strand RNA substrates, a previous research from our laboratory reported the structures of individual KH domains of hMEX3C in complex with RNA fragment, which are the only reported hMEX-3 family protein-RNA complex structure^17^. Based on the structural studies, the authors suggested the RNA substrate motifs recognized by hMEX3C and the bound pattern between the individual KH domains and the corresponding RNA substrates. In the current research, we combined the NMR and SAXS methods and demonstrated tandem KH domains of the hMEX3B in complex with the 3′UTR RNA fragment derived from HLA-A mRNA. We observed conformational change between the RNA-free and RNA-bound proteins. Furthermore, we determined two pairs of threonines and arginines (TRTR) on the KH domains’ variable loops, which play a key role in the recognition between hMEX3B and RNA substrate. We investigated the details of how TRTR mutation affects hMEX3B function and the downstream pathway both in vitro and in vivo. Finally, we concluded that hMEX3B reduced the amount of HLA-A protein on the surface of the tumor cell and promoted tumor cell escape from the immune kill carried out by the T cell.

## Results

### hMEX3B tandem KH domains bind a 12-nt RNA from 3′UTR of HLA-A mRNA

To investigate the mechanism of KHs interacting with their RNA fragments based on the secondary structures of amino acid sequences (Fig. 1b), we expressed three different protein truncations, including individual KH domains, KH1 (56-144) and KH2 (156-230), and tandem KH domains KH1/2 (56-230), using the *E. coli* protein expression system. The oligomerization and homogeneity of the three proteins were verified by gel filtration chromatography and SDS‒PAGE (Supplementary Fig. S1a, S1b). Previous research reported that the RNA elements recognized by ceMEX-3 from *C. elegans* were (A/G/U) (G/U) AGN_0–8_U(U/A/C) UA^18^. In addition, the RNA fragments that can be mediated by hMEX3C thus had common sequencing characteristics: (A/G/U) (G/U) AGN_0-8_ (A/G/U) (A/U) (A/U) (A/G/U)^17^. As the KH domains of the MEX3 family are relatively conserved (Fig. 1b), we searched for potential substrates of hMEX3B using these two RNA motifs in the 3′ UTR of human HLA-A mRNA. As a result, we selected three RNA fragments that matched the basic motifs for the in vitro hMEX3B RNA binding experiments (Fig. 1c and Supplementary Fig. S1c). After synthesizing all three substrates, we carried out Isothermal Titration Calorimetry (ITC) experiments using the KH1/2 protein, and found that the binding affinity between KH1/2 and 12-nt RNA (CUGAGCUGUAAC) was very strong, but the binding modes of KH1/2 with 13-nt or 17-nt RNAs were not unique (Fig. 1d, Supplementary Fig. S1d and S1e). These differences may be caused by the multiple recognition sites in the fragments (different motifs are marked as colored lines in Supplementary Fig. S1d and S1e)^19^. The single K-homology domain typically accommodates 4–5 ribonucleotides in its binding clefts^17,20,21^. The 12-nt substrate, according to the binding pattern, can be divided into two short fragments: UGUAAC, which interacted with KH1, and CUGAGC, which contacted KH2. The ITC results indicated that the individual KH domain of hMEX3B can bind to corresponding 6-nt RNAs with much lower affinities (more than 100 times lower) compared with the longer pairs (Fig. 1e, f). Therefore, the tandem KH domains provided not only the RNA-binding ability but also enough specificity for the recognition of substrate. At the same time, we checked the interactions between the individual KH domains and the 12-nt RNA to eliminate the effect of the differences from the substrates. As expected, the binding affinity of either KH1 or KH2 to the 12-nt RNA was even weaker than that of the 6-nt RNA, while the N value from the fitted curves also indicated that the binding stoichiometry of protein to RNA was close to 2:1 (Supplementary Fig. S1f, S1g and Supplementary Table S1).

### Structural models of hMEX3B individual KH domains with 6-nt RNAs

**Fig. 2:**
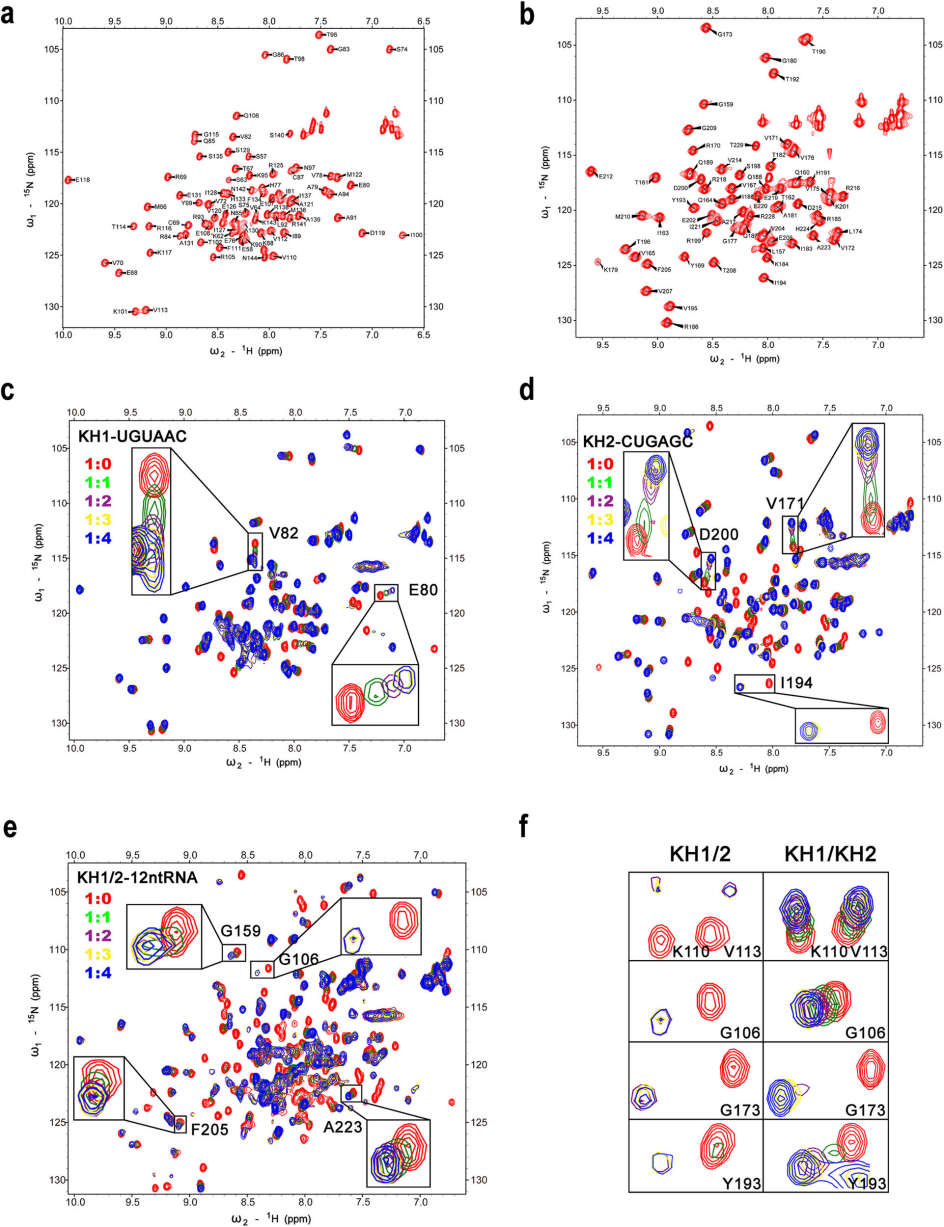
NMR assignments of hMEX3B individual KH domains and the titration of KH proteins with their RNAs. **a**, **b** ^1^H-^15^N HSQC spectrum of hMEX3B individual KH domains with the assignment of backbone chemical shifts. **c**–**e** NMR titration of ^15^N-labeled hMEX3B individual KH domains and KH1/2 domains by indicated RNA fragments. As the RNA concentration increased, the amino acids which have the most obvious chemical shift perturbation are shown in the enlarged squares. **f** The common residues in both hMEX3B individual KH domains and the KH1/2 domains have similar chemical shift disturbances.

To address the details of how hMEX3B contacted the RNA substrates, we carried out structural studies on the individual KH domains in complex with their matching 6-nt RNAs. After failing to generate the complex crystals, we used nuclear magnetic resonance methods to investigate the binding characteristics of the KH-RNA complex with the perdeuterated [^15^N, ^13^C]-labeled proteins. We first expressed and purified the ^13^C and ^15^N double-labeled KH1 and KH2 proteins and recorded a series of three-dimensional spectra, such as CBCANH, CBCA(CO)NH, HNCA, and HN(CO)CA, for amino acid residues assignments. We assigned 79 HN of 89 residues (including 5 prolines) in the 2D ^1^H-^15^N HSQC spectrum of the hMEX3B KH1 domain, with completion of 94.0% (Fig. 2a), and we assigned 62 HN of 75 residues (including 5 prolines) in the 2D ^1^H-^15^N HSQC spectrum of the hMEX3B KH2 domain, with 88.6% completion (Fig. 2b). Furthermore, we carried out NMR chemical shift perturbation experiments to identify the interface between the individual KH domains and 6-nt RNAs. We used different concentrations of 6-nt RNAs to titrate the hMEX3B KH1 (or KH2) proteins. As the molar ratio between KH domains and RNAs increased from 1:0 to 1:4, several amino acids showed obvious shifts, indicating that they were interfered with by the RNA (Fig. 2c, d). By measuring and calculating the chemical shift disturbance of all residues, we mapped all the residues with apparent chemical shift disturbances in the KH domains (Supplementary Fig. S2a and S2b), which are (V72 A79 E80 V82 R84 Q85 C87 K88 A91 A94 Y99 I100 T102 R105) in the KH1 domain and (R170 V171 L174 K179 T182 K184 I186 Q187 V195 I194 S198 D200 H224 R228) in the KH2 domain.

At the same time, we also recorded the 2D HSQC spectra of the hMEX3B KH1/2 protein. By superimposing it on the individual KH1 and KH2 proteins, we could not find any obvious shift peaks except the additional peaks due to the linker region in the KH1/2 protein. This result suggested that the two KH domains did not contact each other when the RNA was absent (Supplementary Fig. S2c). We also used the 12-nt RNA fragments to serially titrate the KH1/2 protein to investigate the interacting information. As the concentration of 12-nt RNA increased, some signals in the KH1/2 domains underwent intense chemical shift disturbances, and some of them even disappeared due to intermediate exchange, such as G106, G159, F205, and A223 (Fig. 2e). After comparing the chemical shifts in the titrating experiments of the individual KHs and KH1/2, we noticed that the perturbed residues were similar; the only difference was that some peaks shift more in the KH1/2 than in the separated KH protein (Fig. 2f). This finding indicated that the binding affinity between KH1/2 and 12-nt RNA was much stronger than the individual KHs with their 6-nt RNAs and matched with the ITC results we mentioned above.

### The complex structural models of two individual KH domains with 6-nt RNAs

**Fig. 3:**
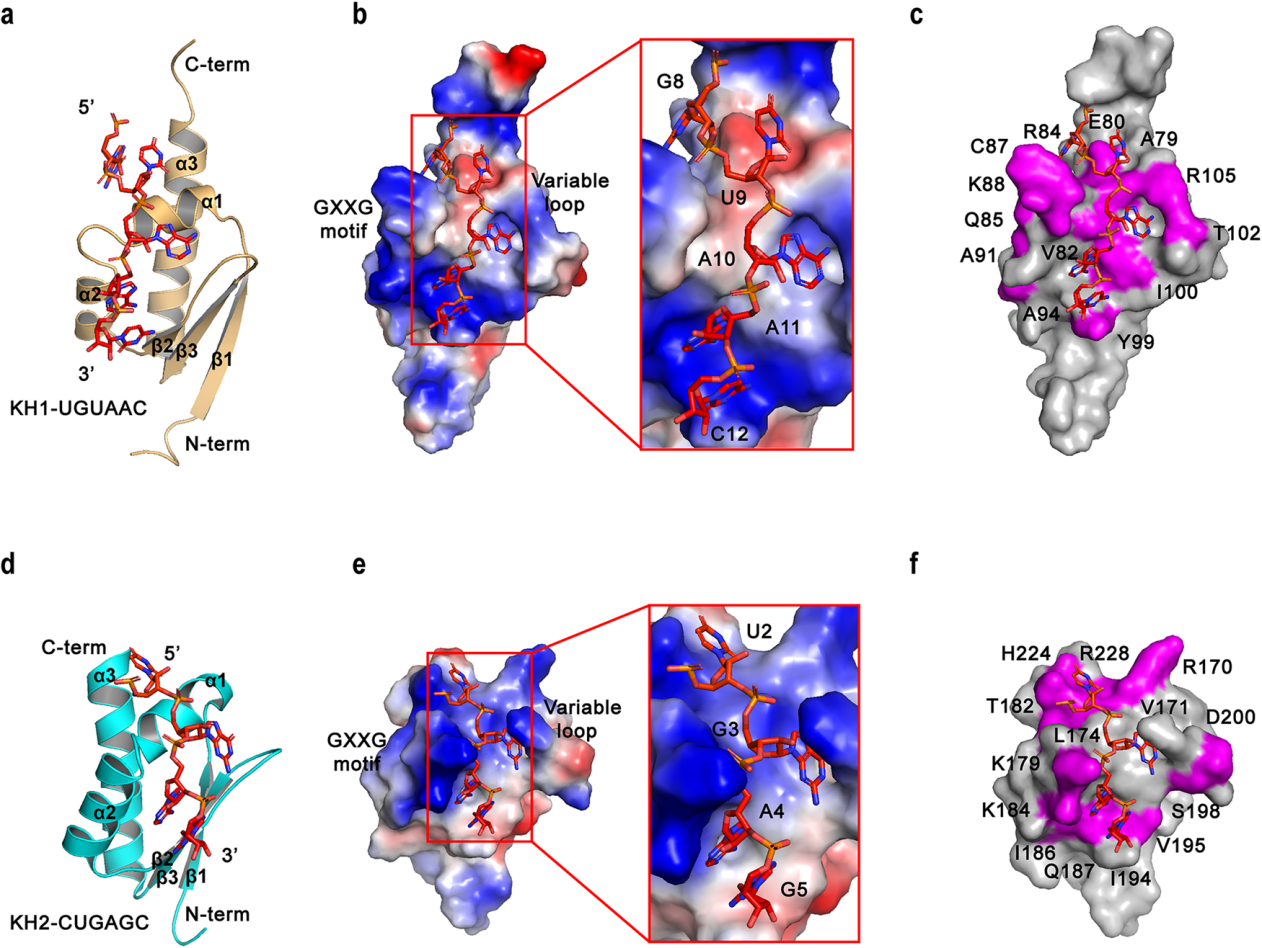
The complex structural models of two individual KH domains with 6-nt RNAs. **a** The hMEX3B KH1 complex structural model is represented as a ribbon model. The KH1 domain is colored yellow, and the 6-nt RNA (UGUAAC) is shown as a stick representation with red color. **b** The electrostatic surface of the KH1-RNA complex surface. Then, electropositive and electronegative regions are shown in blue and red, respectively. **c** The residues (colored magenta) undergoing chemical shift perturbations upon RNA binding were visualized on the surface of the KH1-RNA complex model. **d** The hMEX3B KH2 complex structural model is represented as a ribbon model. The KH2 domain is colored cyan, and the 6-nt RNA (CUGAGC) is shown as a stick representation with red color. **e** The electrostatic surface of the KH2-RNA complex surface. Regions of electropositive and electronegative potential are shown in blue and red, respectively. **f** The residues (colored magenta) undergoing chemical shift perturbations upon RNA binding were visualized on the surface of the KH2-RNA complex model. All the structural figures in this manuscript were generated by PyMOL v2.0.

To further study the details of the interaction between hMEX3B and its RNA substrates, we applied homologous replacement with hMEX3C (hMEX3C KH1-RNA, PDB code: 5WWW; hMEX3C KH2-RNA, PDB code: 5WWX) as the template to generate the hMEX3B individual KH domain models using Chimera X software^22^. After that, we employed HADDOCK to generate models of the individual KH domains in complex with their corresponding 6-nt RNAs based on the interacting residue information obtained from NMR as constraints (Fig. 3)^23^. The two individual KH domains were both classical type I KH domains, in which three α-helices packed against a β-sheet composed of three antiparallel β-strands, their topological arrangement being β-α-α-β-β-α, the GXXG motif is located between α1 and α2, and the variable loop is located between β2 and β3 of the KH domains (Fig. 3a, d).

In both complex structural models, the base of the nucleotide (A11 and C12 for KH1; A4 and G5 for KH2) is anchored by a hydrophobic pocket largely constituting residues from the α2 helix, β2 strand, and variable loop (I89, R93, I100, K101, and T102 for KH1; I183, Q187, I194, V195, and T196 for KH2). The base of the nucleotide (A10 for KH1; G3 for KH2) is anchored by a hydrophobic pocket largely constituting residues from the α1 helix and variable loop (S75, V78, T102, P103, and R105 for KH1; V172, T196, P197, and R199 for KH2) (Fig. 3b, c, e, f).

The 6-nt RNA fragments crossed the middle of α3 near the C-terminus and were inserted into the positively charged grooves formed by α2, β2, the GXXG motifs and the variable loops of the KH domains. In the KH1-RNA model (Fig. 3a–c), the core recognition sequence is UAAC, which occupies 4 RNA binding sites on the KH1 domain, and in the KH2-RNA structure (Fig. 3d–f), the core recognition sequence is UGAG, which occupies four RNA binding sites on the KH2 domain.

### Structural models of the RNA-free and RNA-bound forms of hMEX3B KH1/2

To further illustrate how the hMEX3B KH1/2 protein binds with the 12-nt RNA fragment, we collected small-angle X-ray scattering (SAXS) data of both the KH1/2 domain apo form and the 12-nt RNA-bound form. The SAXS data indicated that both KH1/2 and KH1/2-12-nt RNA complexes formed monomers in the solution. We also carried out size exclusion chromatography multi-angle light scattering experiments (SEC-MALS) to confirm the oligomeric state. As shown in Supplementary Figure S3a, both the apo and RNA-bound forms of KH1/2 existed as monomers in the solution; furthermore, the KH1/2 and 12-nt RNA bound together with a molar ratio of 1:1. When observing the hMEX3B motif on its natural substrate, HLA-A mRNA, we noticed that KH1 recognized the 3′ side 6-nt in the longer RNA substrate, while KH2 bound with the 5′ side (schemed in Supplementary Fig. S3b). Hence, we wondered whether the hMEX3B substrate recognition had the orientation or not. We synthesized the switched 12-nt RNA fragment (5′-UGUAACCUGAGC-3′) and determined the binding affinity by ITC. The Kd between KH1/2 and it was larger than 1.11 μM, which is more than 25 times weaker than the original 12-nt RNA fragment (Supplementary Fig. S3b, S3c). Therefore, this result suggested that the binding of hMEX3B and RNA was not only sequence-specific but also direction dependent.

**Fig. 4:**
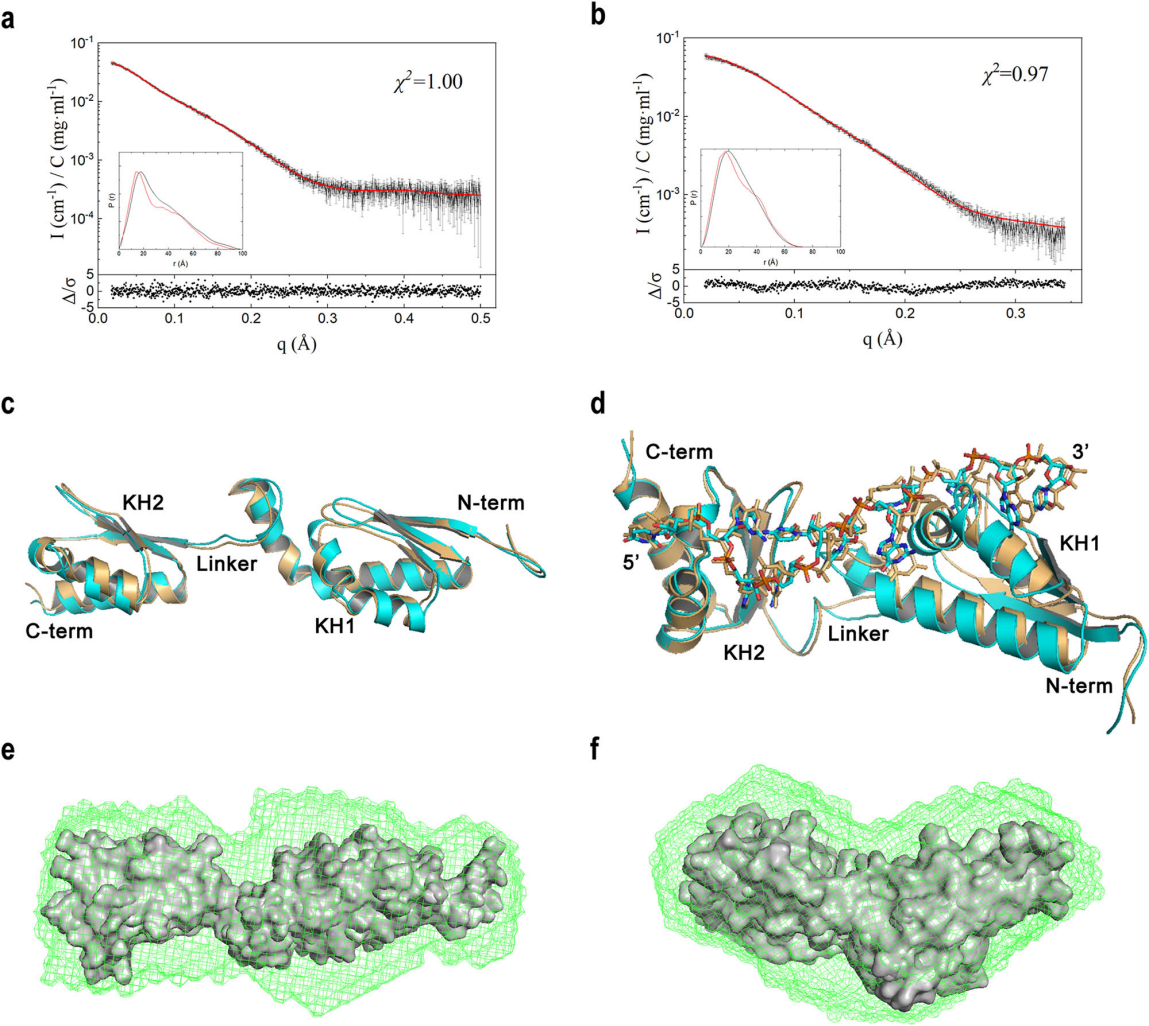
The apo and complex forms of the structures of hMEX3B KH1/2 calculated by MD simulation. **a**, **b** The back-calculated SAXS profile of the selected ensemble (red line) is fitted to the experimental data (black line with errors) of the hMEX3B KH1/2 domains apo form **a** and complex form **b**. The lower plot shows the error-weighted residual of the model fitting. The inset is the PDDF calculated from the selected ensemble (red curve) and SAXS data (black curve). **c**, **d** The models of the hMEX3B KH1/2 apo **c** and complex **d** forms were calculated from the SAXS data by MD simulation. **e**, **f** The two models from Dammif and the two structures (surface) from molecular dynamics simulation were matched well by using supcomb from ATSAS package. The NSDs of the apo form **e** and the complex form **f** were 1.48 and 1.22, respectively.

After we obtained the above information about the binding patterns between hMEX3B and its RNA substrate, we tried to generate the structural model of the KH1/2-12-nt RNA by molecular dynamics (MD) simulation methods. Different from conventional MD simulation, integrative modeling combines an enhanced sampling method and experimental data such as SAXS to efficiently sample the conformations of the apo form and complex proteins^24^. During the simulation of the KH1/2 apo form, we found that χ2 decreases relatively fast in the first five cycles and then slowly converges to approximately 1.0 after the 5th cycle (Supplementary Fig. S4a). The same situation occurred when simulating the KH1/2-12-nt RNA complex (Supplementary Fig. S4b). Therefore, we plotted the calculated SAXS profile of the ensemble at the 5^th^ cycle and its error-weighted residual for both the KH1/2 apo form (Fig. 4a) and RNA-bound form (Fig. 4b). The residuals were defined as (I_exp(q)_ – I_calc(q)_)/σ_exp(q)_, corresponding to the difference between the experimental and the computed intensities weighted by the experimental uncertainty. The residual difference plot was flat, which indicates that the results were in good agreement with the data. The inset shows the normalized average PDDF of the ensemble, which had a similar shape to the experimental PDDF in both models (Supplementary Fig. S4c for the apo form and Supplementary Figure S4d for the complex form). To characterize conformations consistent with the SAXS data, the distribution of Rg values of the apo form (black line) and the bound form (red line) were calculated from the ensembles in the last 10 molecular dynamics cycles. There was a major peak with an Rg value of approximately 21.8 Å for the complex model, and there were two peaks with Rg values of approximately 26.4 Å and 27.2 Å for the apo model (Fig. S4k). The structural models of both peaks of the apo form were aligned and are shown in Fig. 4c, while the models of the major peak of the KH1/2-12-nt RNA complex are shown in Fig. 4d. By comparing the models of RNA-free and RNA-bound forms, we noticed that KH1/2 became more compact after binding with RNA. At the same time, Kratky plots of both also supported that the 12-nt RNA made the KH1/2 more ordered than the apo form (Supplementary Fig. S4e and S4f). In addition, we also used Dammif software to establish the models of the apo form and RNA-bound form using the SAXS data, and the two models from Dammif and the two structures from molecular dynamics simulation were fitted well by calculation with supcomb (NSD for apo form was 1.48 Å and visualized in Fig. 4e; NSD for RNA complex was 1.22 Å and visualized in Fig. 4f), proving that our structural models were very reliable from different aspects^25,26^.

### Double T/R in the variable loops had a great effect on RNA recognition

**Fig. 5:**
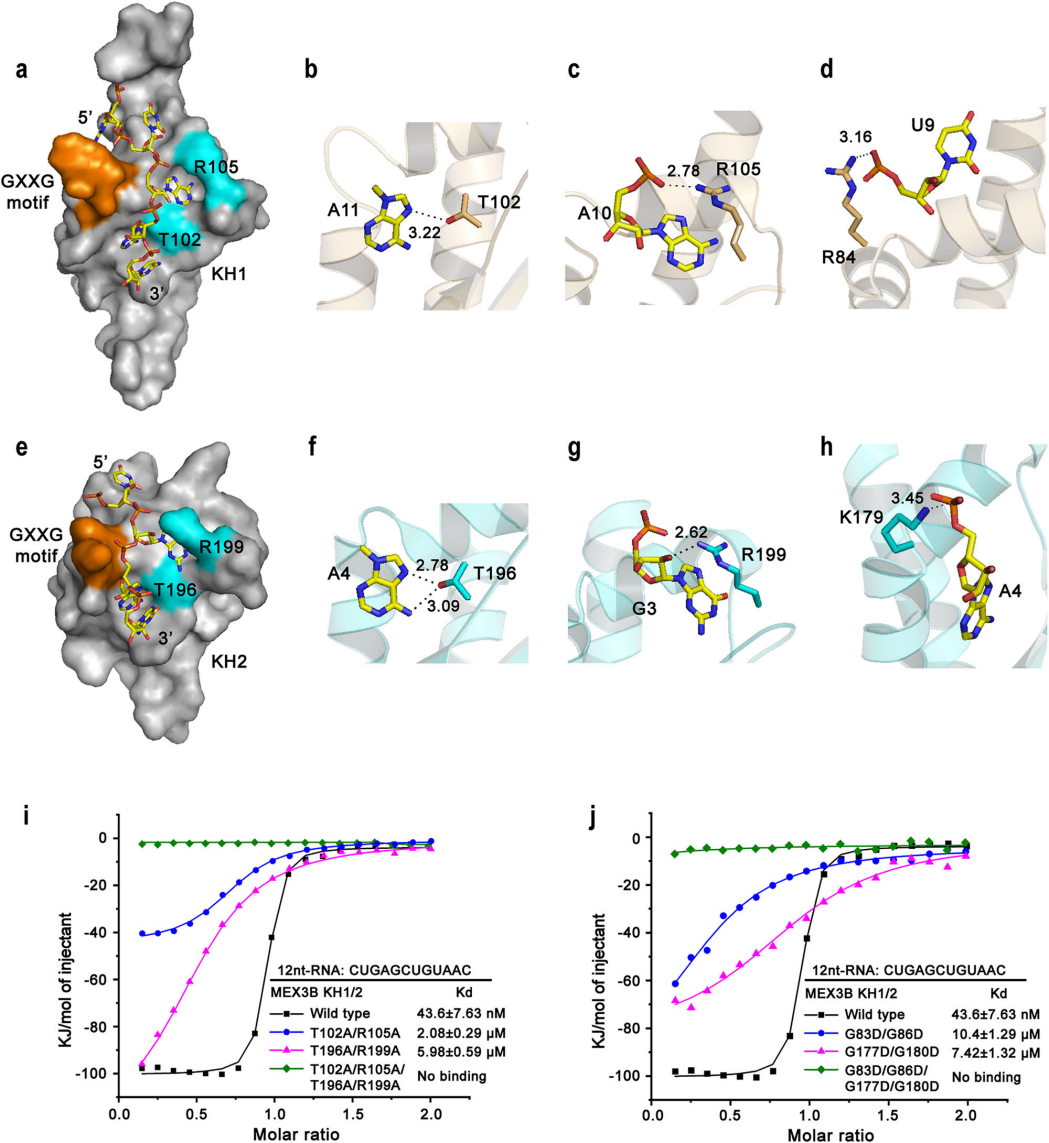
Double T/R in the variable loops greatly affects RNA recognition. **a** The surface models of KH1 in complex with its corresponding RNA. Double T/R were marked in cyan and the GXXG motifs were marked in orange. The RNAs were shown as red ribbons. **b**–**d** The interactions between ribonucleotides and TR regions **b**, **c** or GXXG motif **d** in the KH1 domain. Important interactions (H-bond and/or salt bridge) are depicted as dashed lines. **e** The surface models of KH2 in complex with its corresponding RNA. Double T/R were marked in cyan and the GXXG motifs were marked in orange. The RNAs were shown as orange ribbons. **f**–**h** The interactions between ribonucleotides and TR regions **f**, **g** or GXXG motif **h** in the KH2 domain. **i** The binding affinities of the hMEX3B KH1/2 wild type or three mutants (T102A/R105A, T196A/R199A and TR4A) with 12-nt RNA. **j** The binding affinities of the hMEX3B KH1/2 wild type or three GXXG mutants (G83D/G88D, G177D/G180D and G4D) with 12-nt RNA.

When investigating the structural model of the protein–RNA complex, we noticed that the RNA fragment laid in the positively charged groove consisted of KH1 and KH2 (Supplementary Fig. S5a and S5b). When checking more deeply, we found that the GXXG motifs and the variable loops clamped the RNA molecule like three fingers, one was the GXXG motifs (Orange regions in Fig. 5a, e and Supplementary Fig. S5c), and the other two were the two residues on the variable loops, which were T102 and R105 from KH1 and T196 and R199 from KH2 (cyan residues in Fig. 5a, e and Supplementary Fig. S5c). As there are many studies on the GXXG motif, we mainly focused on the mechanism of how the newly identified TRTR influenced hMEX3B recognition of the RNA substrate and regulated the functions of downstream proteins.

The interactions of protein-RNA were contributed by multiple hydrogen bonds and hydrophobic contacts between the amino acids and the ribonucleotides: the nitrogen in the adenine ring of A11 interacted with the hydroxyl group of the T102 from KH1, while, or two nitrogen atoms in the adenine ring of A4 contacted with the hydroxyl group of the T196 of KH2) (Fig. 5b, f); the phosphate group of A10 and the ribose of G3 connected with the side chain amino groups of the R105 and R199 on the KH1 and KH2, respectively (Fig. 5c, g). In addition, the side chain amino group of R84 within the GXXG motif grips the RNA fragment by forming a hydrogen bond with the phosphate group of A11 in the KH1 complex (Fig. 5d), whereas the side chain amino group of K179 from the same motif of the KH2 domain form hydrogen bonds with the phosphate group of A4, respectively (Fig. 5h).

Based on these facts, we tried to mutate these residues to alanines to explore the effects if we abolished parts of or all of these hydrogen bonds. Previous research found that mutating any one point of these key residues in individual domains reduced the binding affinities^17^. Therefore, we chose some combined mutants and purified three KH1/2 mutants: mutated in KH1 (T102A/R105A), mutated in KH2 (T196A/R199A), and mutant TR4A (referred to T102A/R105A/T196A/R199A), and checked their binding abilities with 12-nt RNA fragments using ITC methods. As expected, when the TR in KH1 (T102A/R105A) or KH2 (T196A/R199A) were mutated separately, the binding affinities, when compared with the wild type, decreased approximately 50 times and 137 times, respectively. However, when we mutated all four key residues together (mutant TR4A), the KH1/2 protein could not bind to 12-nt RNA at all (Fig. 5i). To exclude the structural alteration in the mutation, we carried out circular dichroism with both the wild type and the TR4A mutation, and the results indicated that there was no obvious secondary structural difference between them (Supplementary Fig. S5d). At the same time, we also did the same mutations in GXXG motifs (G83D/G86D in KH1 or G177D/G180D in KH2 or both G4D in KH1/2), and we got similar results as TRTR mutations (Fig. 5j and Supplementary Fig. S5d). Interestingly, similar to the GXXG motif, the TRTR are highly conserved among different MEX-3 proteins from different species according to the sequencing alignments (Fig. 1b). This indicates the key regulations of the TRTR to the function of the MEX-3 family might be as important as the well known GXXG motifs.

Furthermore, we also examined the nucleotides involved in hMEX3B-RNA interactions. We synthesized different RNA fragments that contain different point mutants compared to the original 12-nt RNA we used before and carried out ITC measurements as before. The results illustrated that mutation of any one of the key nucleotides (U2, A4, A10 or A11) severely disrupted the protein-RNA interactions and lowered the binding affinities to twenty times and more (Supplementary Fig. S5e–S5h).

### The mutant TR4A disrupted hMEX3B function in vivo

**Fig. 6:**
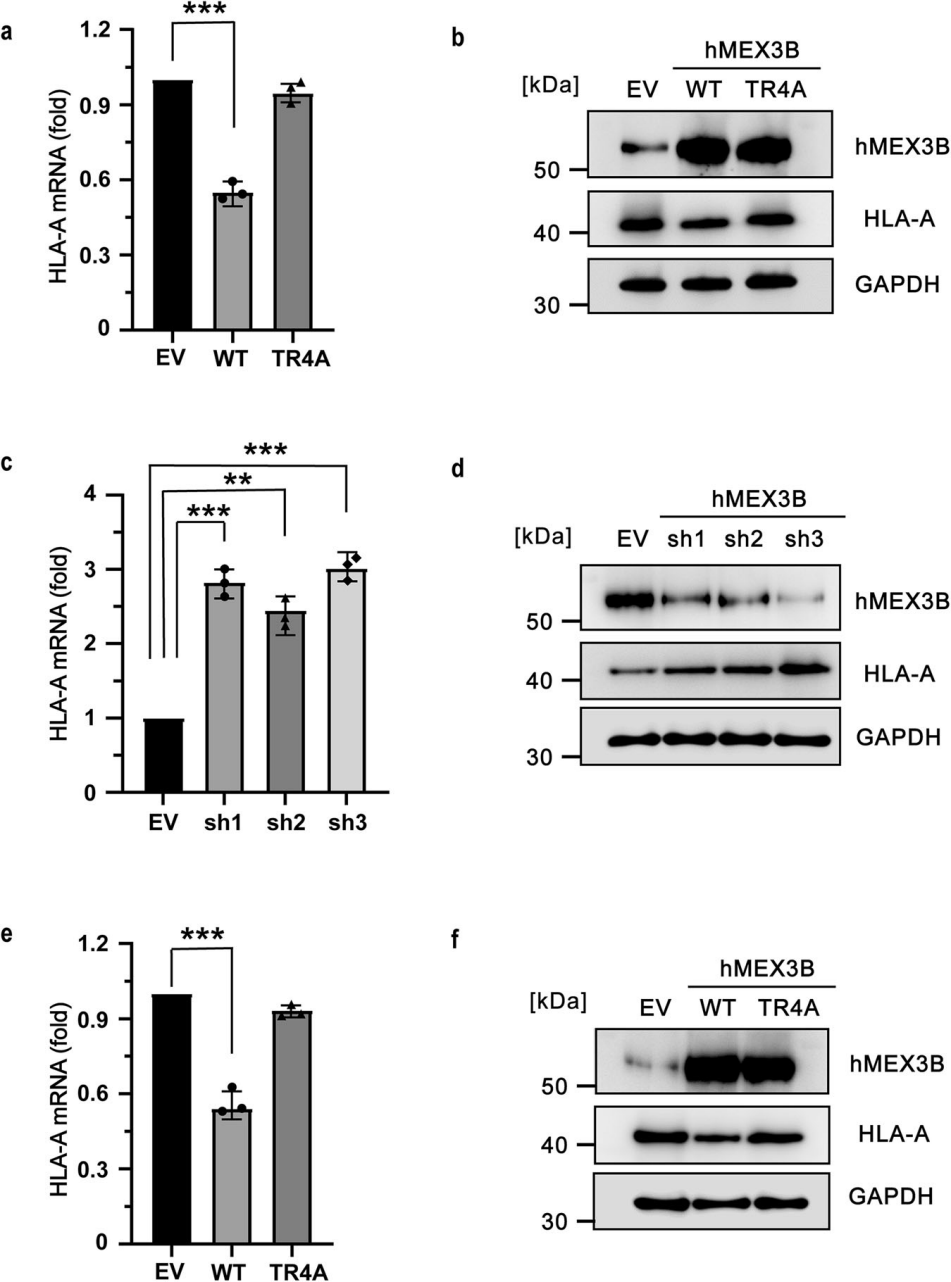
Double T/R is required for hMEX3B function in vivo. **a**, **b** The mRNA levels **a** of HLA-A and the protein levels **b** of hMEX3B and HLA-A in the 293 T cells overexpressed the wild type or TR4A mutant of hMEX3B full-length protein. The control cells were transfected with an empty vector. The protein amount of GAPDH was used as sample amount control in all western blotting in this article. **c**, **d** The mRNA levels **c** of HLA-A and the protein levels **d** of hMEX3B and HLA-A in the 293 T cells transfected with three different shRNAs. **e**, **f** The mRNA levels **e** of HLA-A and the protein levels **f** of hMEX3B and HLA-A in the hMEX3B-KD 293 T cells, whose endogenous hMEX3B were knocked down with sh3, complemented with the wildtype or TR4A mutant. All mRNA results were obtained from three independent experiments and the *p* value was unpaired two-tailed t test (*p < 0.05; **p < 0.01).

**Fig. 7:**
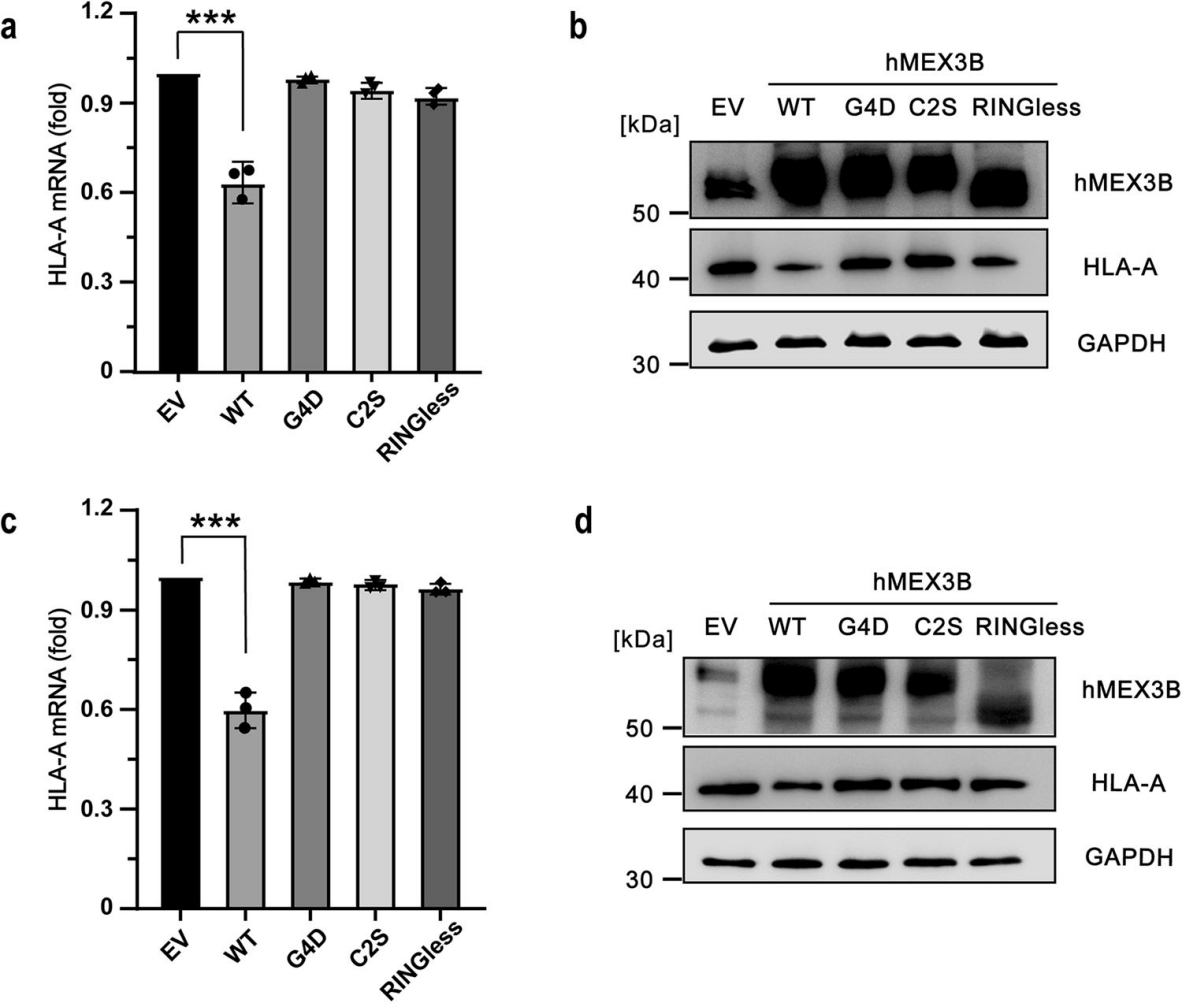
The deficiency of HLA-A caused by other hMEX3B mutations. **a**, **b** The mRNA levels **a** of HLA-A and the protein levels **b** of hMEX3B and HLA-A in the 293 T cells overexpressed the wild type, G4D (mutations in the GXXG motifs), C2S (mutations on the key residues of RING domain) and RINGless (truncation that didn’t contain the RING domain) of hMEX3B full-length protein. **c**, **d** The mRNA levels **c** of HLA-A and the protein levels **d** of hMEX3B and HLA-A in the hMEX3B-KD 293 T cells which were rescued with wildtype, G4D, C2S, and RINGless mutants of hMEX3B full-length protein. The control cells were transfected with an empty vector. The protein amount of GAPDH was used as a sample loading control. All mRNA results were obtained from three independent experiments and the *p-value* was unpaired two-tailed t test (**: *p* < 0.01; ***: *p* < 0.001).

As we investigated the mechanism of how hMEX3B recognized its RNA substrate and the influence of the TR4A mutations on the interaction between hMEX3B and RNA in vitro, we wanted to determine whether TR4A affected the function of hMEX3B in vivo as severely as in vitro. We have discussed before that previous research reported that hMEX3B bound to the 3’UTR of HLA-A mRNA, intermediated the degradation of the mRNA, decreased the expression of HLA-A protein on the surface of tumor cells, and increased the tumor’s immune escape ability. Therefore, we tried to overexpress full-length hMEX3B containing the TR4A mutations in 293 T cells to check the consequence of the mutation in the tumor cells following a previous research setup^11,27,28^. After transfection, the mRNA level of hMEX3B (TR4A) was at the same level as that in the hMEX3B (WT) control group (Supplementary Fig. S6a), but the mRNA level of HLA-A in the cells expressing hMEX3B (TR4A) was higher than that in the cells expressing hMEX3B (WT) and close to that in the cells containing the empty vector as a control (Fig. 6a). Western blotting confirmed this result (Fig. 6b). Furthermore, we intended to eliminate the effect of the endogenous hMEX3B protein, so we tried to use three different shRNAs to knockdown endogenous hMEX3B. The results indicated that all three genes behaved well, especially, the sh3 had the best knockdown effect among them (Fig. 6c, d, and Supplementary Fig. S6b). As the substrate of hMEX3B, HLA-A in all three shRNA-infected groups remained high amount compared with the control group, especially the sh3 group. As the effects of the three shRNAs were similar, we chose to use sh3 to construct the hMEX3B knockdown (hMEX3B-KD) cell line for further study. On top of it, we overexpressed hMEX3B (TR4A) and hMEX3B (WT) in hMEX3B-KD cells. After we checked the mRNA levels and the protein levels, the results were more conspicuous, showing that 293 T cells expressed much less HLA-A by overexpressing hMEX3B (WT), and the mutant 4 A mutations abolished this effect by abrogating the RNA binding ability of the KH1/2 region (Fig. 6e, f, and Supplementary Fig. S6c). In addition, we checked the effect of GXXG motifs on the HLA-A. The hMEX3B G4D contains the four glycine mutations in the GXXG motifs expressed in the 293 T cell, the results indicated G4D will increase both the RNA and the protein levels of HLA-A due to the loss-of-function of hMEX3B mutant (Fig. 7a, b, and Supplementary Fig. S7a). The effect of the mutation in the GXXG motifs is similar to the TRTR mutation pointing out that the TRTR region we newly reported in this manuscript (to the best of our knowledge) was the same important as the well-known GXXG motifs. On the other hand, we also checked the phenotype of 293 T cell which overexpressed the RING domain deficient hMEX3B constructs: C2S which comprehends two key cystines in the RING domain and RINGless refers to the hMEX3B truncated the C terminal RING domain. The results illustrated that both C2S and RINGless mutants could abolish the HLA-A regulation caused by hMEX3B as the TRTR mutations (Fig. 7a, b, and Supplementary Fig. S7a). Beyond this, we also carried out the dominant negative experiments by overexpressing the G4D, C2S, or RINGless in the hMEX3B-KD cells which endogenous hMEX3B was knocked down with the sh3. The results proved similar conclusions that all three deficient mutations can suppress the degradation of HLA-A caused by the hMEX3B on both RNA and protein levels (Fig. 7c, d, and Supplementary Fig. S7b). Combined all together, the hMEX3B recognized the RNA substrate through its tandem KH domains and mediated the degradation of it with the RING domain.

## Discussion

**Fig. 8:**
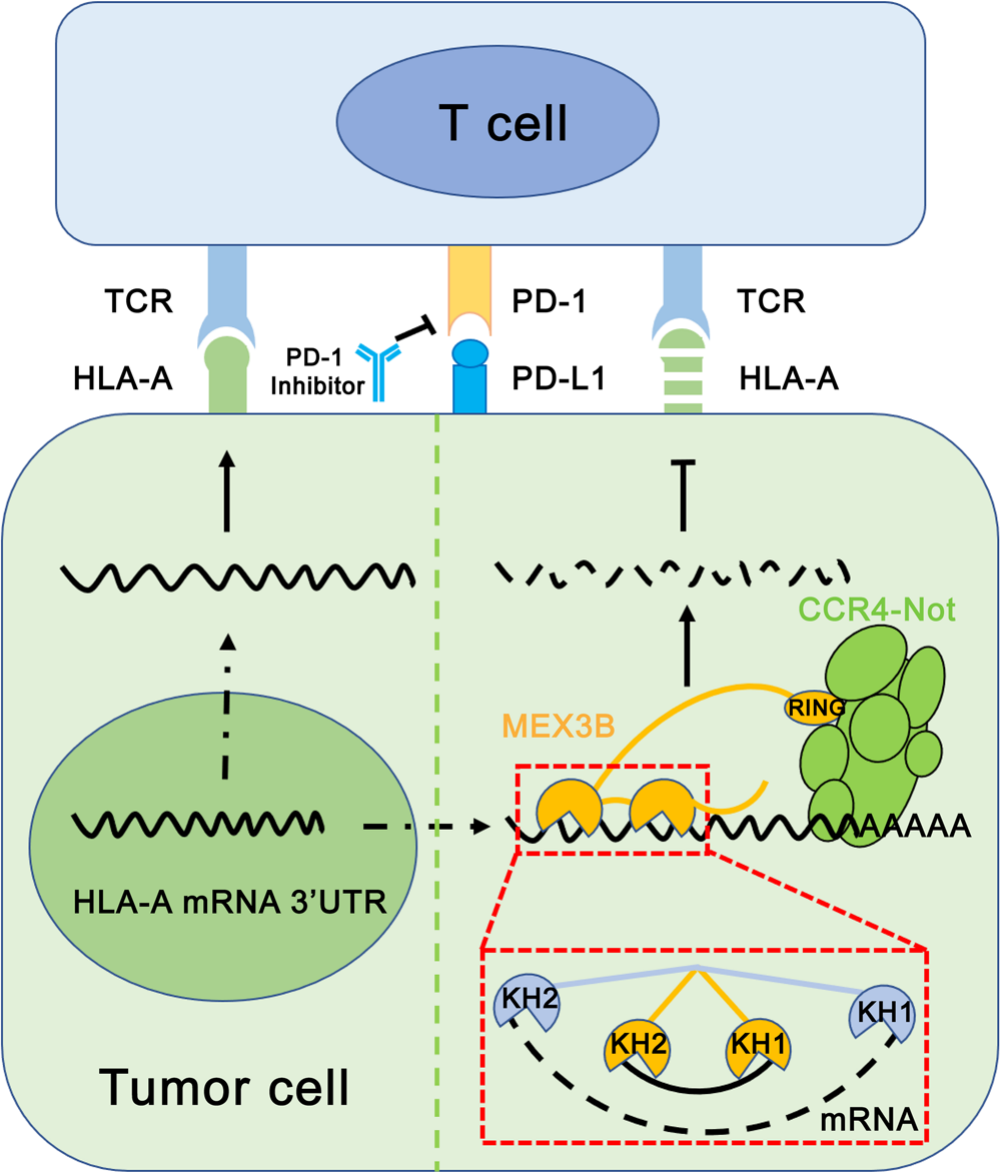
A model of hMEX3B function in the tumor escape during the TCR immune therapy. A model demonstrated how hMEX3B, as a negative effector of anti-PD-1 antibody therapy, reduced the HLA-A amount by degrading its mRNA to increase the immune escape ratio of the tumor cells in detail.

Although it has been found that hMEX3B can regulate the translation of many mRNAs, HLA-A is the only mRNA that has been experimentally confirmed to be regulated by hMEX3B protein^11^. Unlike its homolog in *C. elegans*, in addition to the N-terminal tandem KH domains that take charge of recognizing HLA-A mRNA, hMEX3B also has a RING domain at its C-terminus, which has E3 ligase activity. The CCR4-NOT complex, which has both deadenylation and ubiquitination activities, is composed of eight protein components; two subunits among them (CNOT6/6 L and CNOT7/8) have deadenylase activities, while the others are scaffolds and regulating proteins. The hMEX3B RING domain recruits the CCR4-NOT complex, through the interaction between RING and CNOT2/3 heterodimer, to the target mRNA recognized by the KH domains. After deadenylation, the target mRNAs are degraded by 5′-3′ exoribonuclease 1 and 2 through the exonuclease pathway^29–34^. In tumor cells, HLA-A is located outside the cell membrane and presented as an antigenic substance. T lymphocytes can recognize the HLA-A molecule and activate the immune response with the T-cell receptor (TCR). On the other hand, tumor cells adopt another pathway to defend against this killing process through the PD-L1/PD-1 axis formed by programmed cell death protein-1 (PD-1) and its natural ligand programmed cell death ligand-1 (PD-L1)^35–37^. hMEX3B, as a negative effector of anti-PD-1 antibody therapy, reduces the HLA-A amount by degrading its mRNA to increase the immune escape ratio of the tumor cells (Fig. 8). We detailedly and systematically examined the characteristics of how hMEX3B interacted with its substrate RNA, especially the necessity of two KH domains working spontaneously and coordinately. In particular, we narrowed the key residues that disrupted the interactions to 4 amino acids (TRTR), which may provide a new window leading to a potential gene therapy targeting hMEX3B, especially for patients who failed PD-1 antibody therapy (Figs. 6 and 7).

In the cell, the RNA binding proteins (RBPs) and their RNAs always form the phase separating condensation to proceed with the RNA highly efficiently. Recent research also mentioned that ceMEX-3 can form RNP granules in the oocytes of *C. elegans* but is isolated from the other phases in the P-granule to handle the RNA individually^38^. The C-terminus of ceMEX-3 is the potential phase separation driver based on the sequence, while there is a similar long intrinsically disordered region in hMEX3B connecting the KH domains and RING domain. Interestingly, hMEX3B has the longest linker among all four homologs in humans. The physical fundamentals for phase separation are the multivalent interactions between RBPs and RNAs^39^. As we tested before, except for the best sequence, which we used as a 12-nt RNA, the 3′ UTR of HLA-A mRNA also has another two sites (13-nt and 17-nt) that can bind with hMEX3B in multiple binding modes. Therefore, on one mRNA, multiple hMEX3B molecules bind to and form a separated condensate to recruit multiple CNOT complexes and degrade the mRNA quickly and precisely. The hMEX3B immune fluorescent images in other literature also show the same phenotype in the cytoplasm, so this will be another attractive direction that can be examined later.

## Methods

### Cloning, protein expression, and purification

The full length *hmex3b* gene (Gene ID: 84206) was synthesized from Life Technologies. The DNA segments expressing the human hMEX3B KH1 domain (residues 56-144), KH2 domain (residues 156–230), and tandem KH1/2 domains (residues 56–230) were amplified by PCR. Subsequently, the KH1 and KH2 domains were cloned into a modified pET28a (originally purchased from Novagen, cat# 69864) vector without the thrombin cleavage site, whereas the tandem KH domains were cloned into a modified pET28a vector in which the thrombin cleavage site was substituted with a small ubiquitin-like modifier (SUMO) protease cleavage site. The mutants of the KH1/2 domains were constructed by conventional PCR using the MutanBEST kit (Cat# R401, TaKaRa Biomedical Tech.) and verified by DNA sequencing.

All recombinant proteins were expressed in *E. coli* strain BL21 (DE3) cells (Novagen) cultured in LB medium to an OD600 of 1.0 and then induced with 0.03 mM isopropyl β-D-1-thiogalactopyranoside (IPTG) for 24 h at 16 °C. The cell pellets expressing the target protein were lysed with sonication on the ice and the supernatant was collected and on the nickel-chelating column (Cat# 30210, Qiagen) using binding buffer (2 M NaCl, 20 mM Tris, pH7.5). All recombinant proteins were further purified by size-exclusion chromatography using a HiLoad 16/600 Superdex 75 pg column (Cat# 90100805, Cytiva Life Science) equilibrated with SEC buffer (2 M NaCl, 20 mM Tris, pH7.5). Finally, all the wild type and mutants proteins were treated with ULP1 protease (purified by our lab) to cleave the 6xHis-SUMO tags and following gel filtration was carried out to remove the protease and tag proteins.

### Nuclear magnetic resonance

The ^15^N, ^13^C-labeled hMEX3B KH1, KH2, and KH1/2 proteins used for NMR experiments were expressed by growing the bacteria in LeMaster and Richards medium (2.5 g/L D-Glucose [Cat# CLM-1396-PK, Cambridge Isotope Laboratories, Inc.], 0.5 g/L ^15^NH_4_Cl [Cat# NLM-467-PK, Cambridge Isotope Laboratories, Inc.], 24 g/L KH_2_PO_4_, 5 g/L NaOH, 100 μM CaCl_2_, and 2.2 mM MgSO_4_) and purified by the same procedure as described above. Backbone resonances of the KH1 and KH2 domains were assigned using 0.5 mM ^15^N,^13^C-labeled protein in NMR buffer (50 mM sodium phosphate, pH 6.5, 100 mM NaCl, 5 mM TCEP, 10% D_2_O [Cat# DLM-7005, Cambridge Isotope Laboratories, Inc.]) using CBCANH, CBCA(CO)NH, HNCA, and HN(CO)CA on a Bruker DMX 600 MHz at 298 K. All NMR data were processed using NMR Pipe^40^, and the spectra were assigned using Sparky^41^. The chemical shift assignments for KH1 and KH2 have been deposited in BioMagResBank (http://www.bmrb.wisc.edu) under accession numbers 52147 and 52148, respectively.

### NMR chemical shift perturbations

NMR HSQC spectra were acquired at 298 K on either an Agilent 700 MHz spectrometer equipped with a cryoprobe. The ^15^N-labeled KH1, KH2, and KH1/2 domains (each at a concentration of 0.1 mM) in NMR buffer were titrated with increasing amounts of 6-nt RNA (5′-UGUAAC-3′), 6-nt RNA (5′-CUGAGC-3′), and 12-nt RNA (5′-CUGAGCUGUAAC-3′), respectively. A series of 2D ^1^H, ^15^N HSQC spectra were acquired at the molar ratio between RNA and protein increasing from 0 to 4. The NMR data were processed as above.

### The structural models of KH1-RNA and KH2-RNA complexes

HADDOCK is an information-driven docking technique used for modeling biomolecule structures by using experimental or predictive restraints. The CSPs, obtained from the NMR HSQC titration data, were used both as HADDOCK restraints and for defining the protein active residues. The initial models of hMEX3B KH1 and KH2 were generated by residue substitutions using the crystal structures of the hMEX3C corresponding domains (PDB codes: 5WWW for KH1 and 5WWX for KH2). The docking calculations were done by the HADDOCK web server (https://alcazar.science.uu.nl/services/HADDOCK2.2)^42^.

### Small-angle X-ray scattering (SAXS)

SAXS data were collected in beamline BL19U2 of the Shanghai Synchrotron Radiation Facility (SSRF). Sample concentrations were different from 1 to 5 mg/ml. Referring runs with buffers were performed multiple times and used for buffer subtractions. Measurements were carried out as technical triplicates in four to ten frames to enable the exclusion of data in the case of radiation damage. Data were processed and analyzed with the ATSAS package version 2.8, including the plot of paired-distance distribution, P(r), the determination of D_max_ and R_g_, and theoretical scattering curves^43^. The KH1/2 protein model and KH1/2-12-nt RNA model from theoretical scattering curves were calculated with Dammif^26^.

### Molecular dynamics simulations

In this work, all atom conventional MD (cMD) simulations and accelerated MD (aMD) simulations were conducted using the Amber20 packages^44^.

The cMD was performed with A19SB force field for protein and OL3 force field for RNA^45^ in combination with 4-point OPC water model^46^. The apo form and the complex were built via the LEaP module. The OPC water molecules were added to a truncated octahedral box with a minimal distance of $15.0\,{{{\text{\AA }}}}$ between the solute and box boundary. ${{{{\rm{Na}}}}}^{+}$ and ${{{{\rm{Cl}}}}}^{-}$ ions were added to balance the charge on the protein and bring the salt concentration to about 150 mM. For the apo form, the box size is $9.69\times {10}^{5}\,{{{\text{\AA }}}}^{3}$, with 117,192 atoms in total. For the complex, the box size is $1.01\times {10}^{6}\,{{{\text{\AA }}}}^{3}$, with 121,848 atoms in total. To remove bad contacts, the water molecules and ions were initially minimized for 5000 steps using the steepest descent method for the first 3000 steps and then the conjugate gradient for 2000 steps, with the position of solute fixed (force constant was $500.0\,{{{\rm{kcal}}}}\cdot {{{{\rm{mol}}}}}^{-1}\cdot {{{\text{\AA }}}}^{-2}$). In the second energy minimization, the restraints on the solute were removed. This stage was conducted for 5000 steps, using the steepest descent method for the first 3000 steps and then the conjugate gradient algorithm for 2000 steps. After energy minimization, a heat-up MD was run at a constant volume. The systems were heated from 0 K to 300 K in 100 ps with a weak restraint of $10.0\,{{{\rm{kcal}}}}\cdot {{{{\rm{mol}}}}}^{-1}\cdot {{{\text{\AA }}}}^{-2}$ on the solute. Then free MD simulations of 100 ns were carried out under NPT conditions utilizing the GPU-accelerated pmemd.cuda code. The temperature was regulated using the Langevin dynamics with a collision frequency of $1.0\,{{{{\rm{ps}}}}}^{-1}$^47^. Pressure was controlled with isotropic position scaling at 1 bar with a relaxation time of $2.0\,{{{\rm{ps}}}}$. All the bonds involving hydrogen atoms were constrained using SHAKE algorithm^48^. The step size was $2\,{{{\rm{fs}}}}$. The long-range electrostatic interaction was calculated using the PME method^49^ with a $10\,{{{\text{\AA }}}}$ cutoff for the short-range nonbonded interaction.

aMD^50^ introduces a bias potential that in practice reduces the height of local barriers, allowing the system to evolve faster than cMD. In aMD, the modification of the potential is defined by the following equation

1 $${V}_{{bias}}\left(r\right)=\left\{\begin{array}{c}V\left(r\right),V\left(r\right)\ge E\\ V\left(r\right)+\Delta V\left(r\right),V\left(r\right) \, < \, E\end{array}\right.$$

2 $$\Delta V\left(r\right)=\frac{{({E}_{p}-V(r))}^{2}}{{\alpha }_{p}+{E}_{p}-V\left(r\right)}+\frac{{\left({E}_{d}-{V}_{d}\left(r\right)\right)}^{2}}{{\alpha }_{d}+{E}_{d}-{V}_{d}\left(r\right)}$$

where $V\left(r\right)$ is the normal potential and ${V}_{d}\left(r\right)$ is the normal torsion potential. ${E}_{p}$ and ${E}_{d}$ are the average potential and torsion energy, respectively. The terms ${\alpha }_{p}$ and ${\alpha }_{d}$ are factors that determine inversely the strength with which the boost is applied. With the modification, the deeper regions of the potential energy surface will rise proportionally, while the higher barriers will lower, which as a result makes the potential energy surface flatter but still preserves the underlying shape. To estimate the parameters of aMD, we performed a 100 ns cMD simulation for the apo form and the complex, respectively (Supplementary Figures S4g to S4j). For the apo form, we have ${E}_{p}=-354076\,{{{\rm{kcal}}}}\cdot {{{{\rm{mol}}}}}^{-1},{\alpha }_{p}=18571\,{{{\rm{kcal}}}}\cdot {{{\rm{mo}}}}{{{{\rm{l}}}}}^{-1},{E}_{d}=1637\,{{{\rm{kcal}}}}\cdot {{{{\rm{mol}}}}}^{-1},{\alpha }_{p}=140\,{{{\rm{kcal}}}}\cdot {{{{\rm{mol}}}}}^{-1}$. For the complex, ${E}_{p}=-369919\,{{{\rm{kcal}}}}\cdot {{{{\rm{mol}}}}}^{-1},{\alpha }_{p}=19496\,{{{\rm{kcal}}}}\cdot {{{\rm{mo}}}}{{{{\rm{l}}}}}^{-1},{E}_{d}=1982\,{{{\rm{kcal}}}}\cdot {{{{\rm{mol}}}}}^{-1},{\alpha }_{p}=148.8\,{{{\rm{kcal}}}}\cdot {{{{\rm{mol}}}}}^{-1}$. All the other parameters were the same as those in the cMD simulations.

### SAXS-oriented ensemble refinement

The aMD explores different regions of the conformational space, but we only need those that match the SAXS data. Therefore, we can use the SAXS data to determine which regions to explore. We have developed a method called SAXS-oriented ensemble refinement (SAXS-ER)^51^, and the steps are as follows.

1. Set up the system starting from an initial structure and perform a preliminary aMD simulation of 300 ps.
2. Calculate the discrepancy between the experimental data and the structures generated in the simulation. Find the best fitting ensemble which contains ${N}_{{es}}$ different representative conformations. The representative conformations are picked out from the input structures. The ensemble optimization method^52^, which will be introduced below, was used in this step.
3. Starting from the ${N}_{{es}}$ conformations selected in step 2, ${N}_{{es}}$ independent simulations are carried out to generate new structures. Multiple independent short-time simulations may achieve a better sampling than a single long-time simulation.
4. Repeat steps 2 and 3 for N cycles.

A structure ensemble was obtained by the ensemble optimization method (EOM)^52^. With a given ensemble, the scattering intensity curve, $I\left(q\right)$, can be calculated.

3 $$I\left(q\right)=\frac{1}{N}\mathop{\sum }\limits_{n=1}^{N}{I}_{n}\left(q\right)$$

where $N$ is the number of representative conformations in the ensemble, and ${I}_{n}\left(q\right)$ is the calculated scattering intensity of *n*^th^ representative conformation. ($q=\frac{4{{{\rm{\pi }}}}\sin \theta }{\lambda }$, where $2\theta$ is the scattering angle and $\lambda$ is the wavelength)

The discrepancy between the experimental and calculated curves is defined as follows

4 $${\chi }^{2}=\frac{1}{K-1}\mathop{\sum }\limits_{i=1}^{K}{\left[\frac{\mu I\left({q}_{i}\right)-{I}_{{exptl}}\left({q}_{i}\right)}{\sigma \left({q}_{i}\right)}\right]}^{2}$$

where $K$ is the number of experimental points, $\sigma \left(q\right)$ are standard deviations, and $\mu$ is a scaling factor. By minimizing ${\chi }^{2}$, the final ensemble that best fits the experimental SAXS data will be found. In this work, we used EOM2 program^53^ and chose the all-atom structural model to perform ensemble optimization.

### Circular Dichroism measurements

Far-UV Circular Dichroism (CD) spectra of the hMEX3B KH1/2 domain and its mutants were determined using an Applied Photophysics Chirascan spectrometer at 298 K. The spectra were recorded at wavelengths between 195 and 260 nm using a 0.05 cm path length cell. The protein samples were diluted to 0.2 mg/ml with CD buffer (30 mM PBS, pH 6.5, 100 mM NaCl). A buffer-only reference was subtracted from each curve. All samples were tested in triplicate.

### Isothermal titration calorimetry

RNA oligomers were synthesized from Takara Bio, Inc. and dissolved in diethyl pyrocarbonate-treated water to a final concentration of 5 mM. The ITC experiments were performed at 298 K using a MicroCal PEAQ-ITC. The proteins and the RNAs were dialyzed in the same buffer as described above. The titration measurements were performed via a single initial injection of 1 μl, followed by 18 injections of 2 μl of different proteins into the sample cell containing different RNAs. In the control experiment, the buffer without protein was injected into the RNA to compensate for the heat of protein dilution. Curve fitting to a single binding site model was performed by the MicroCal PEAQ-ITC analysis software.

### Size exclusion chromatography with multi-angle light scattering (SEC-MALS)

A DAWN HELEOS-II (Wyatt Technology) multi-angle bright scattering detector and an Optilab T-rEX differential refractometer (Wyatt Technology) were used inline with a Superdex 75 Increase 10/300 GL (Cat# 29148721, Cytiva Life Science) column. hMEX3B (2 mg/ml) and 12-nt RNA were mixed at a molar ratio of 1:1.2 in the SEC-MALS buffer (20 mM Bis-Tris, pH 6.5, 150 mM NaCl and 2 mM DTT) and incubated for 2 hours on ice before the experiment. The samples were run at a flow rate of 0.4 ml/min in the SEC-MALS buffer at room temperature. The data were analyzed using ASTRA 6.1 software.

### Plasmids and cell lines

HEK293T cells were cultured in Dulbecco’s modified Eagle’s medium (DMEM) supplemented with 10% (v/v) fetal bovine serum and 1% penicillin/streptomycin. Cells were grown at 37 °C in a humidified atmosphere containing 5% CO_2_. Trypan blue exclusion was used to assess cell viability.

All short hairpin RNAs (shRNAs) against hMEX3B (sequences listed in Supplementary Table S2) were inserted into PLKO.1 vector. Genes encoding hMEX3B and hMEX3B mutants were subcloned into the pcDNA3.1-myc-His vector.

### Western blotting

Cell lysates were prepared in radioimmunoprecipitation assay buffer (50 mM Tris–HCl, pH 8.0, 150 mM NaCl, 5 mM EDTA, 0.1% SDS, and 1% NP-40) supplemented with Roche protease inhibitor cocktails (Cat# 4693132001, Sigma-Aldrich). SDS-PAGE separated equal amounts of total cell lysate. The amount of GAPDH (Cat# of anti-GAPDH is HRP-60004, Proteintech Group Inc.) served as a loading control. Primary antibodies against the following proteins were used in this manuscript: hMEX3B (Cat# WL3452579, Invitrogen), and HLA-A (Cat# DF6890, Affinity). Horseradish peroxidase-conjugated anti-rabbit or anti-mouse (Cat# STAR121 or AAC10P, Bio-Rad Laboratories Inc.) secondary antibodies were used to detect primary antibodies, and the signal was detected using LAS4000 (Cytiva Life Science).

### Real-time fluorescence quantitative PCR (qPCR)

Total RNA from HEK293T cells was extracted using TRIzol (Cat# 15596-026, Invitrogen) solution and reverse transcribed into cDNA for real-time fluorescence quantitative PCR. The RT-qPCR experiments were carried out with a Roche LC96 temperature gradient fluorescence quantitative PCR instrument and using SYBR Green I (Cat# S7585, Thermo Fisher Scientific) as the fluorescent dye, with GAPDH as an internal reference (all primer sequences were listed in Supplementary Table S3).

### Statistics and reproducibility

All the experiments were performed at least three times independently. All values are given as a mean of n ± s.d. *P* values were calculated by unpaired two-tailed t-test from the mean data of each group. All data reported here are reproducible.

### Reporting summary

Further information on research design is available in the Nature Portfolio Reporting Summary linked to this article.

### Supplementary information

Supplementary Materials

Description of Additional Supplementary Files

Supplementary Data 1

Reporting Summary

## Data Availability

The chemical shift assignments for KH1 and KH2 have been deposited in BioMagResBank (http://www.bmrb.wisc.edu) under accession numbers 52147 and 52148, respectively. Supplementary Fig. S8 contains the original uncropped blot/gel images of the main figures and supplementary figures. Supplementary Data 1 contains the source data for the graphs in the figures (main Figs. 6 and 7 and Supplementary Figs. S6 and S7). The other data supporting the findings of this study are available from the corresponding author upon reasonable request.
