# Supplementary material for: Molecular mechanism of specific HLA-A mRNA recognition by the RNA-binding-protein hMEX3B to promote tumor immune escape
Source: Commun Biol. 2024 Feb 7;7:158. doi: 10.1038/s42003-024-05845-y (PMC10850505; doi:10.1038/s42003-024-05845-y)
Supplement: Supplementary file 1 — Supplementary Materials [file 42003_2024_5845_MOESM1_ESM.pdf]

Molecular mechanism of specific HLA-A mRNA recognition by the RNA-binding-protein hMEX3B to promote tumor immune escape

Kanglong Yang[1, 2, 3], Guanglin Chen[4], Fan Yu[1, 2, 3], Xianyang Fang[5], Jiahai Zhang[1, 2, 3], Zhiyong Zhang[4, #], Yunyu Shi[1, 2, 3, #], Liang Zhang[1, 2, 3, #]

1. Hefei National Research Center for Cross disciplinary Science, School of Life Sciences, Division of Life Sciences and Medicine, University of Science and Technology of China, Hefei, Anhui, 230027, P. R. China.

2. Ministry of Education Key Laboratory for Membraneless Organelles and Cellular Dynamics, University of Science & Technology of China, Hefei, Anhui, 230027, P. R. China.

3. Center for Advanced Interdisciplinary Science and Biomedicine of IHM, University of Science & Technology of China, Hefei, Anhui, 230027, P. R. China.

4. Department of Physics, University of Science and Technology of China, Hefei, Anhui 230026, P. R. China.

5. Beijing Advanced Innovation Center for Structural Biology, School of Life Sciences, Tsinghua University, Beijing, 100084, P. R. China.

*To whom correspondence should be addressed. Liang Zhang, Tel: +86 551 63600441; Fax: +86 551 63601 443; Email: zhangl99@ustc.edu.cn

Correspondence may also be addressed to Yunyu Shi. Tel: +86 551 63607464; Fax: +86 551 63601443; Email: yyshi@ustc.edu.cn or Zhiyong Zhang, Tel: +86 551 63606134;Email: zzyzhang@ustc.edu.cn

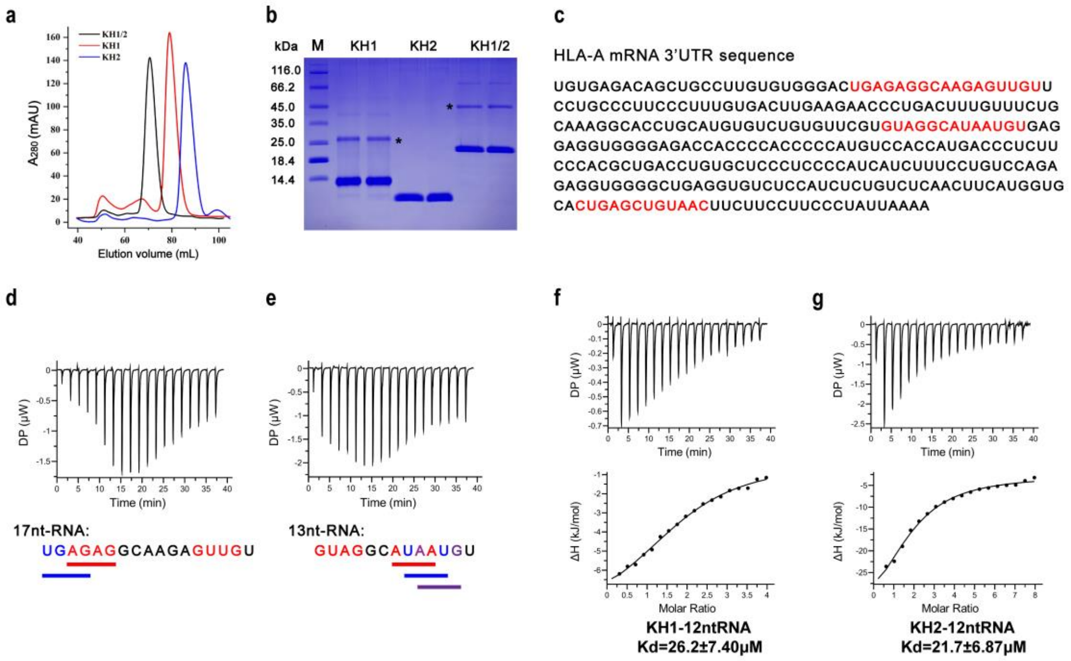

Supplementary Figure S1: (a & b) Protein expression and purification. Less than 5% altered protein bands in KH1 and KH1/2 protein samples (marked with stars in Supplementary Figure S1b) might represent the dimeric forms due to the unstable di-sulfur bond. (c) The full-length sequence of HLA-A mRNA 3'UTR. Sequences marked in red represent three RNA regions containing the hMEX3 family binding motif. (d & e) The ITC results of hMEX3B KH1/2 protein with 17-nt RNA and 13-nt RNA. The lines under the sequence indicate the potential RNA motif. (f & g) The ITC fitting results of individual hMEX3B KH1 (f) or KH2 (g) domains with 12-nt RNA.

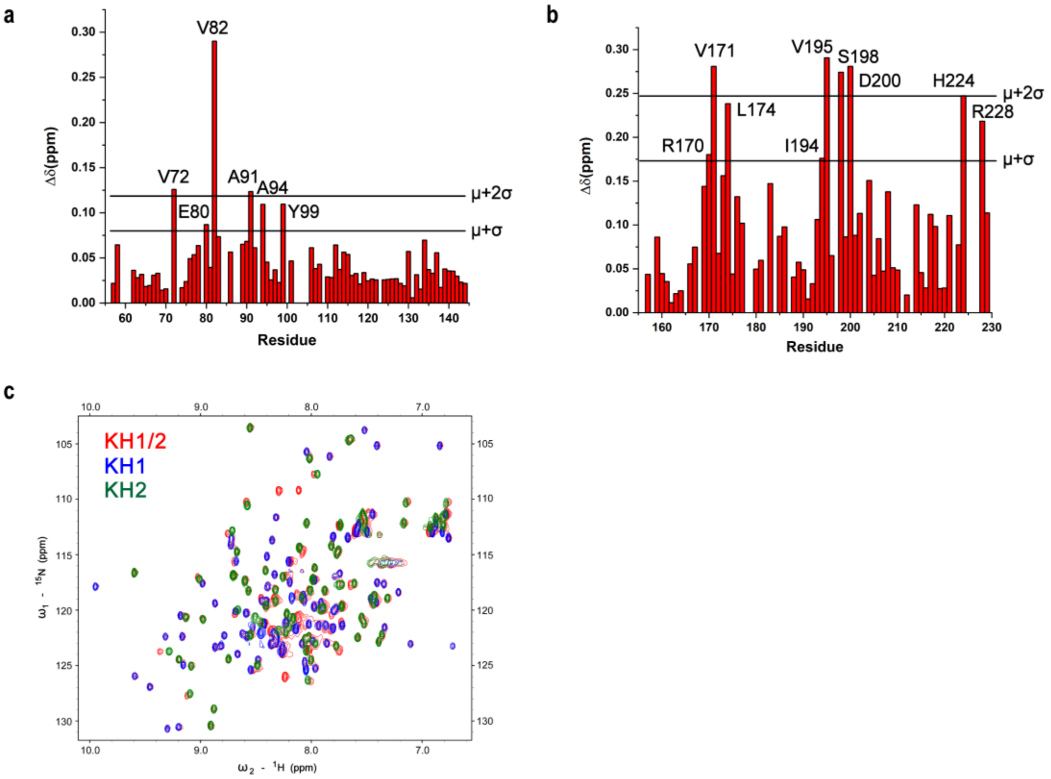

Supplementary Figure S2: (a & b) CSP of the individual KH1 (a) and KH2 (b) domains of hMEX3B. The two lines represent two times (top, μ+2σ) or one time (bottom, μ+σ) standard deviations higher than the average of the chemical shift perturbations. (c) The overlapped HSQC spectra of the individual KH domains and the tandem KH1/2 domains of hMEX3B. The spectra of KH1, KH2 and KH1/2 were colored blue, green and red, respectively. The peaks that only existed in the KH1/2 sample corresponded to the residues on the linker between KH1 and KH2 domains.

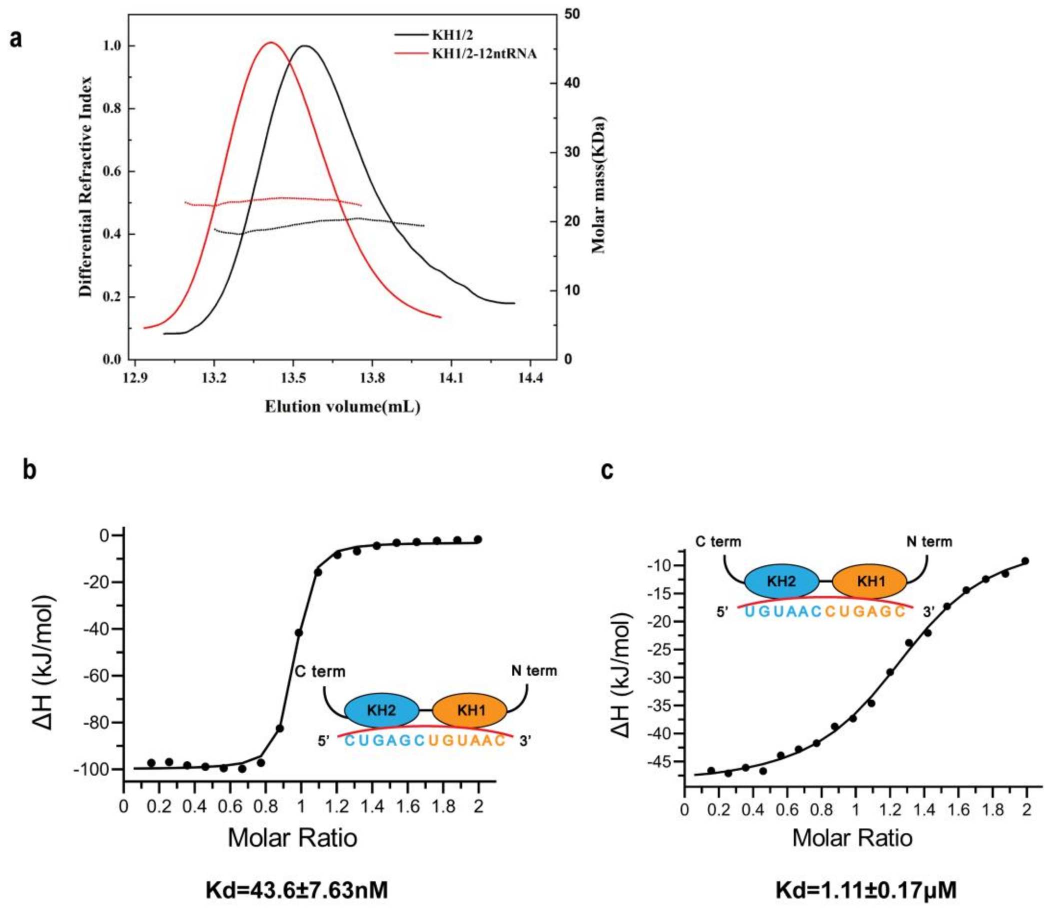

Supplementary Figure S3: (a) The SEC-MALS results of the KH1/2 protein alone (black) and in complex with 12-nt RNA (red). (b & c) The binding affinities between hMEX3B KH1/2 domains with 12-nt RNA (b) or the switched RNA substrate (c) were determined by ITC. The schematic representations of the 12-nt RNA and the switched RNA were shown in panels b and c, respectively.

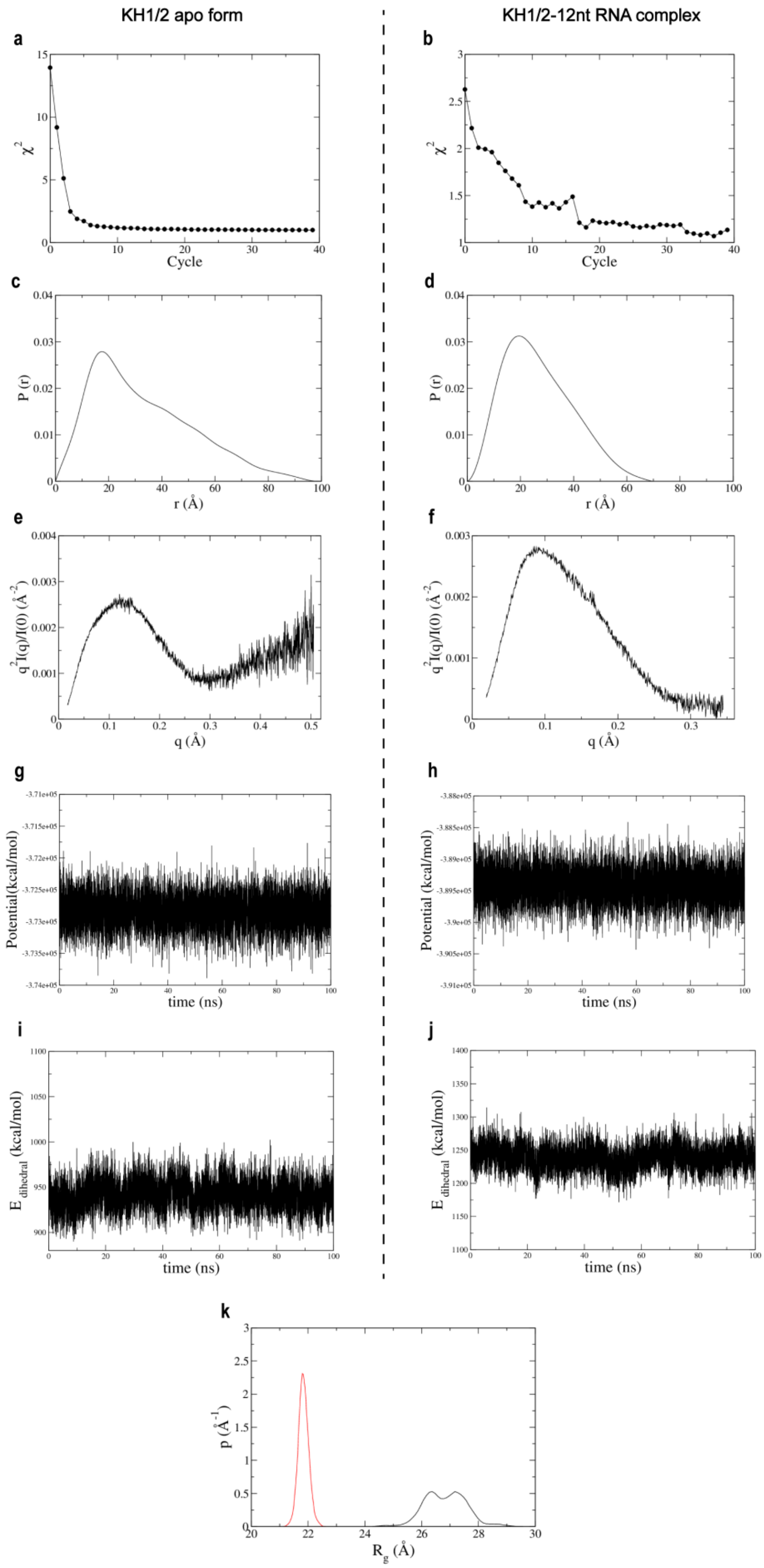

KH1/2 apo form
KH1/2-12nt RNA complex
a
b
χ^2
Cycle
c
d
P (r)
r (Å)
e
f
$q^2I(q)/I(0)$ (Å^{-2})
q (Å)
g
h
Potential(kcal/mol)
Potential (kcal/mol)
time (ns)
i
j
$E_{dihedral}$ (kcal/mol)
time (ns)
k
p (Å^{-1})
R_g (Å)

Supplementary Figure S4: (a & b) The minimal χ^2 of the apo (a) or complex (b) forms at each MD cycle. (Both simulations were carried our triple times individually) (c & d) The pair distance distribution functions (PDDF) of the apo (c) or complex (d) forms calculated from the SAXS data, which were normalized so that the sum under the curve is 1. (e & f) Kratky plots of the apo (e) or complex (f) forms calculated from the SAXS data. (g & h) The total potential of the simulations for the apo (g) or complex (h) forms. (i & j) The dihedral angular energies of the simulations for the apo (i) or complex (j) forms. (k) The distribution of R_g values of the apo (black line) or complex (red line) forms was calculated from the ensembles in the last 10 MD cycles. The data are normalized so that the sum under the curve is 1.

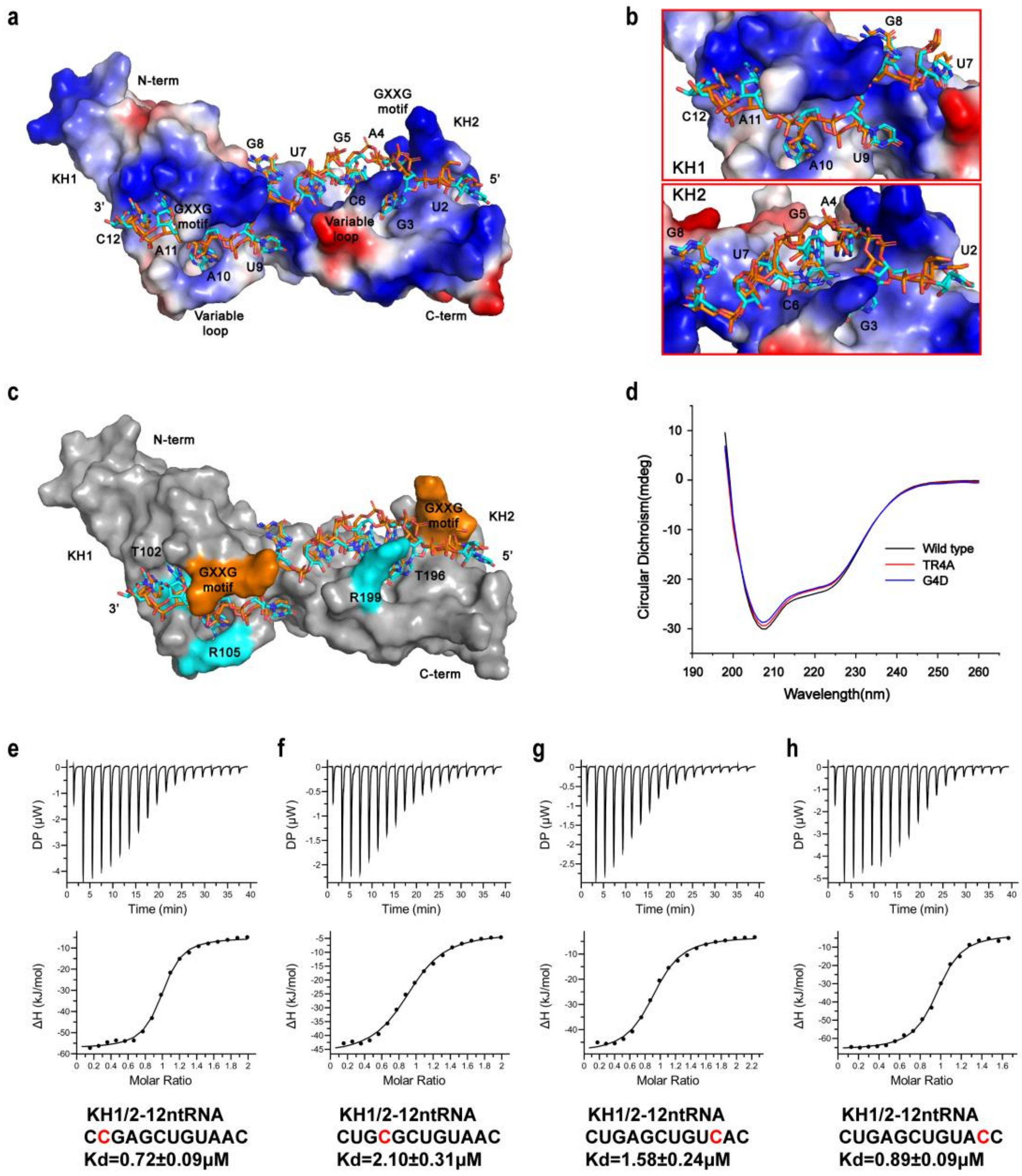

Supplementary Figure S5: (a) The electrostatic surface potential of the KH1/2-12-nt RNA complex. 12-nt RNA is shown as ribbon. Regions of electropositive and electronegative potential were represented in blue and red, respectively. (b) The enlarged details of the KH domains interacting with the RNA. (c) The surface of the KH1/2-12-nt RNA complex. The protein was displayed as a grey surface and the RNA was shown as ribbon, while the GXXG motifs and TRTR regions were colored orange and cyan, respectively. The orientation of panel C is the same as panel A. (d) CD results of the wild type (black), TR4A mutant (red) and G4D mutant (blue) of hMEX3B KH1/2 proteins. (e~h) ITC results of the hMEX3B KH1/2 protein with 12-nt RNAs containing different nucleotide mutants. The different mutant points are marked in red beneath the correlative ITC curves.

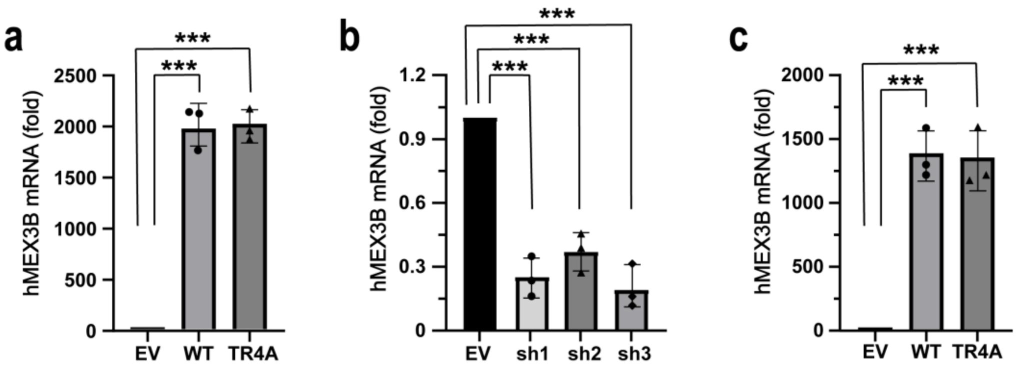

Supplementary Figure S6: (a) RNA levels of hMEX3B variants in the 293T cell overexpressed the wildtype or TR4A mutant of hMEX3B full-length proteins. The control cells were transfected with an empty vector. (b) RNA level of endogenous hMEX3B in the 293T cells transfected with three different shRNAs. (c) RNA levels of hMEX3B variants in the hMEX3B-KD 293T cells rescued with the wildtype and TR4A mutant hMEX3B after knocking down the endogenous hMEX3B with sh3. All mRNA results were obtained from three independent experiments and the *p* value was unpaired two-tailed t test (***: p<0.001; ****: p<0.0001).

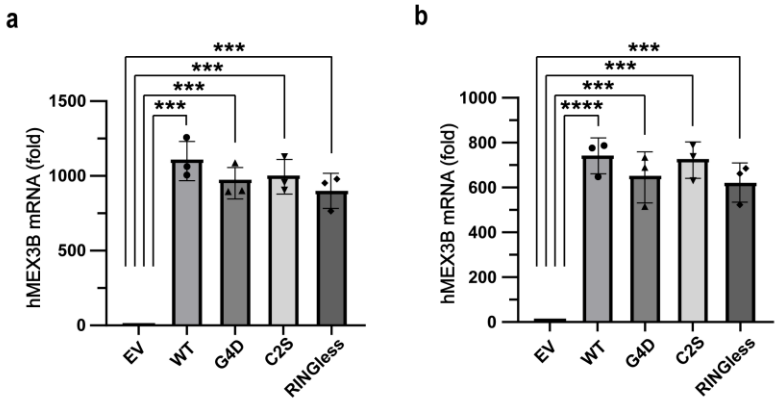

Supplementary Figure S7: (a) RNA levels of hMEX3B variants in the 293T cell overexpressed the wildtype, G4D, C2S or RINGless mutants of hMEX3B full-length proteins. The control cells were transfected with an empty vector. (b) RNA levels of hMEX3B variants in the hMEX3B-KD cells rescued with the wildtype, G4D, C2S or RINGless mutants of hMEX3B. All mRNA results were obtained from three independent experiments and the *p* value was unpaired two-tailed t test (***: *p*<0.001; ****: *p*<0.0001).

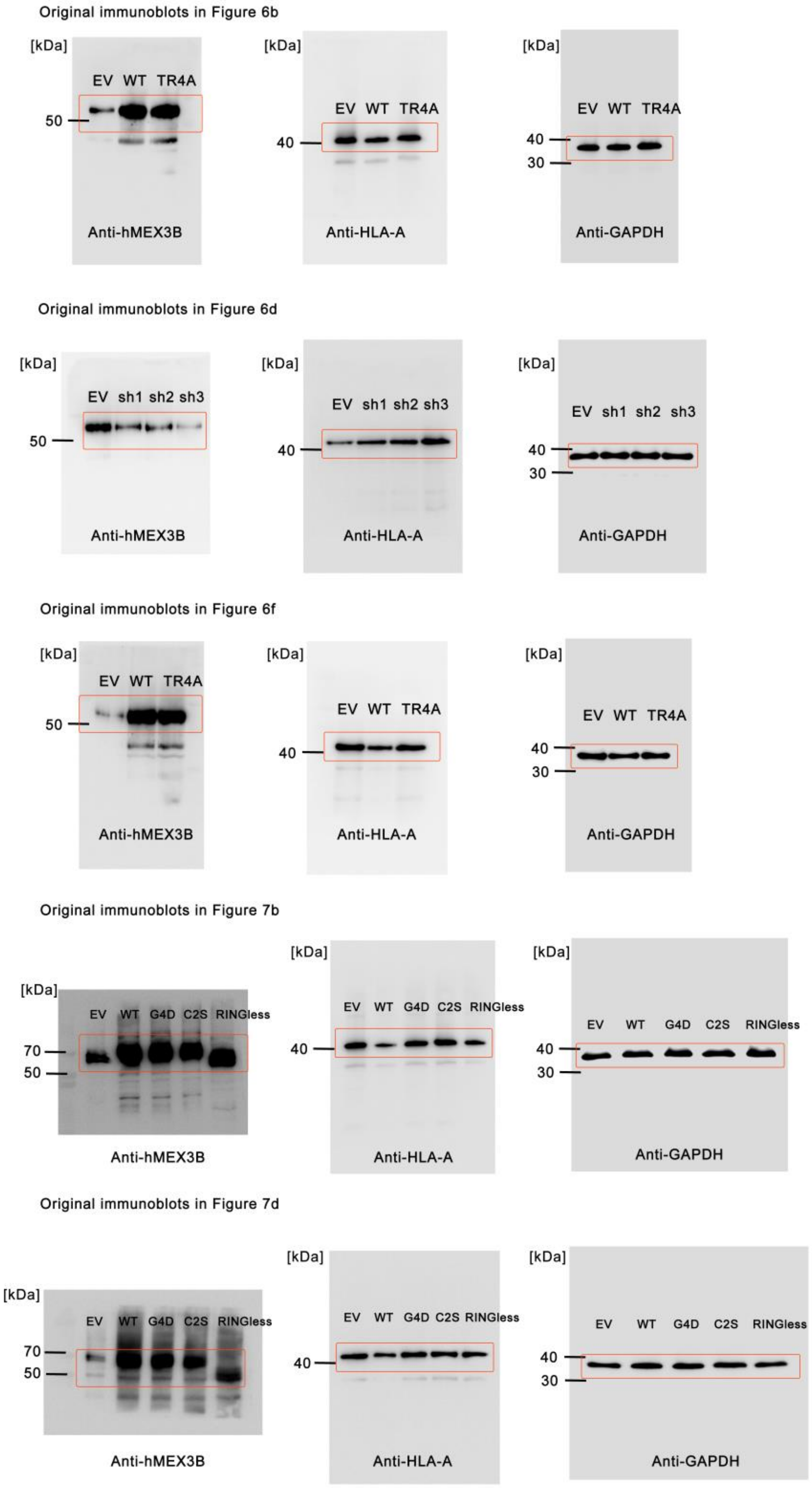

Supplementary Figure S8: Uncropped and unedited blot/gel images for the main figures and supplementary figures.

Supplementary Table S1. The binding affinities of different hMEX3B mutants and RNA substrates.

Protein	RNA Sequence (5’→3’)	Binding affinity (μM)	N value
KH1/2 WT	CUGAGCUGUAAC	0.0436 ± 0.00763	0.91
	UGUAACCUGAGC	1.11 ± 0.17	0.84
KH1 WT	UGUAAC	10.3±0.61	0.89
	CUGAGCUGUAAC	26.2±7.40	1.91
KH2 WT	CUGAGC	9.57±0.99	1.02
	CUGAGCUGUAAC	21.7±6.87	1.79
KH1/2 (T102A/R105A)	CUGAGCUGUAAC	2.08±0.29	0.79
KH1/2 (T196A/R199A)		5.98±0.59	0.68
KH1/2 (TR4A)		No binding	NA
KH1/2 (G83D/G86D)		10.4±1.29	0.67
KH1/2 (G177D/G180D)		7.42±1.32	0.89
KH1/2 (G4D)		No binding	NA
KH1/2 WT	CCGAGCUGUAAC	0.72±0.09	0.96
	CUGCGCUGUAAC	2.10±0.31	0.91
	CUGAGCUGUCAC	1.58±0.24	0.89
	CUGAGCUGUACC	0.89±0.09	0.94
KH1/2 WT	CCCCCCCCCCCC	No binding	NA
KH1 WT	CCCCCC	No binding	NA
KH2 WT	CCCCCC	No binding	NA

Supplementary Table S2. The sequences of shRNAs used in this research

shRNA	Sequence (5’→3’)
sh1	CCGGGCCTTTAACCTCATGGTCAAACTCGA GTTTGACCATGAGGTTAAAGGCTTTTTG
sh2	CCGGCGGTATCTTCTTCCTGCTCTTCTCGAG AAGAGCAGGAAGAAGATACCGTTTTTG
sh3	CCGGGCGCAAGAAGAGCGTGAACATCTCG AGATGTTCACGCTCTTCTTGCGCTTTTTG

Supplementary Table S3. The Q-PCR primers sequences used in this research

Target gene	sequence (5’→3’)	
hMEX3B	Forward Sequence	GAAGAGCGTGAACATGACCGAG
	Reverse Sequence	CCGTCACAACAAAGACAGGCTC
HLA-A	Forward Sequence	AGATACACCTGCCATGTGCAGC
	Reverse Sequence	GATCACAGCTCCAAGGAGAACC
GAPDH	Forward Sequence	TCCCATCACCATCTTCCAGG
	Reverse Sequence	ATGAGTCCTTCCACGATACC
