# Supplementary material for: Molecular mechanism of specific HLA-A mRNA recognition by the RNA-binding-protein hMEX3B to promote tumor immune escape
Source: Commun Biol. 2024 Feb 7;7:158. doi: 10.1038/s42003-024-05845-y (PMC10850505; doi:10.1038/s42003-024-05845-y)
Supplement: Supplementary file 2 — Description of Additional Supplementary Files [file 42003_2024_5845_MOESM2_ESM.pdf]

Description of Additional Supplementary Files

File name: Supplementary Data 1

Description: The source data for the graphs in the figures (main figures 6 and 7 and supplementary figures S6 and S7).
