# Supplementary material for: Molecular mechanism of specific HLA-A mRNA recognition by the RNA-binding-protein hMEX3B to promote tumor immune escape
Source: Commun Biol. 2024 Feb 7;7:158. doi: 10.1038/s42003-024-05845-y (PMC10850505; doi:10.1038/s42003-024-05845-y)
Supplement: Supplementary file 4 — Reporting Summary [file 42003_2024_5845_MOESM4_ESM.pdf]

nature portfolio

Corresponding author(s): Liang Zhang, COMMSBIO-23-2391B

Last updated by author(s): Jan 23, 2024

Reporting Summary

Nature Portfolio wishes to improve the reproducibility of the work that we publish. This form provides structure for consistency and transparency in reporting. For further information on Nature Portfolio policies, see our Editorial Policies and the Editorial Policy Checklist.

Statistics

For all statistical analyses, confirm that the following items are present in the figure legend, table legend, main text, or Methods section.

n/a	Confirmed	
☐	☒	The exact sample size (*n*) for each experimental group/condition, given as a discrete number and unit of measurement
☐	☒	A statement on whether measurements were taken from distinct samples or whether the same sample was measured repeatedly
☐	☒	The statistical test(s) used AND whether they are one- or two-sided<br>*Only common tests should be described solely by name; describe more complex techniques in the Methods section.*
☒	☐	A description of all covariates tested
☒	☐	A description of any assumptions or corrections, such as tests of normality and adjustment for multiple comparisons
☐	☒	A full description of the statistical parameters including central tendency (e.g. means) or other basic estimates (e.g. regression coefficient) AND variation (e.g. standard deviation) or associated estimates of uncertainty (e.g. confidence intervals)
☐	☒	For null hypothesis testing, the test statistic (e.g. *F*, *t*, *r*) with confidence intervals, effect sizes, degrees of freedom and *P* value noted<br>*Give P values as exact values whenever suitable.*
☒	☐	For Bayesian analysis, information on the choice of priors and Markov chain Monte Carlo settings
☒	☐	For hierarchical and complex designs, identification of the appropriate level for tests and full reporting of outcomes
☒	☐	Estimates of effect sizes (e.g. Cohen's *d*, Pearson's *r*), indicating how they were calculated

Our web collection on statistics for biologists contains articles on many of the points above.

Software and code

Policy information about availability of computer code

Data collection	MicroCal PEAQ-ITC control, Chirascan, ASTRA 6.1, Image Quant LAS4000, CFX Maestro
Data analysis	NMR Pipe, Sparky, HADDOCK web server 2.2, Amber20 package, MicroCal PEAQ-ITC analysis, ASTRA 6.1, Pymol, Chimera, GraphPad Prism, Chirascan data viewer, ATSAS-2.8.4

For manuscripts utilizing custom algorithms or software that are central to the research but not yet described in published literature, software must be made available to editors and reviewers. We strongly encourage code deposition in a community repository (e.g. GitHub). See the Nature Portfolio guidelines for submitting code & software for further information.

Data

Policy information about availability of data

All manuscripts must include a data availability statement. This statement should provide the following information, where applicable:

- Accession codes, unique identifiers, or web links for publicly available datasets
- A description of any restrictions on data availability
- For clinical datasets or third party data, please ensure that the statement adheres to our policy

The data underlying this article are available in the article and in its online supplementary material.

Research involving human participants, their data, or biological material

Policy information about studies with human participants or human data. See also policy information about sex, gender (identity/presentation), and sexual orientation and race, ethnicity and racism.

Reporting on sex and gender	This study doesn't include human data
Reporting on race, ethnicity, or other socially relevant groupings	This study doesn't include human data
Population characteristics	This study doesn't include human data
Recruitment	This study doesn't include human data
Ethics oversight	This study doesn't include human data

Note that full information on the approval of the study protocol must also be provided in the manuscript.

Field-specific reporting

Please select the one below that is the best fit for your research. If you are not sure, read the appropriate sections before making your selection.

☒ Life sciences ☐ Behavioural & social sciences ☐ Ecological, evolutionary & environmental sciences

For a reference copy of the document with all sections, see nature.com/documents/nr-reporting-summary-flat.pdf

Life sciences study design

All studies must disclose on these points even when the disclosure is negative.

Sample size	The sample size determined in this study follows general method and there is no predetermined size was used to exclude the oversized sample
Data exclusions	There is no data was excluded during the data collection and analysis
Replication	All the data were replicable
Randomization	All the data were grouped randomly during the data collection and analysis
Blinding	The investigators were blinded during the data collection and analysis

Reporting for specific materials, systems and methods

We require information from authors about some types of materials, experimental systems and methods used in many studies. Here, indicate whether each material, system or method listed is relevant to your study. If you are not sure if a list item applies to your research, read the appropriate section before selecting a response.

Materials & experimental systems

n/a	Involved in the study	
☐	☒	Antibodies
☐	☒	Eukaryotic cell lines
☒	☐	Palaeontology and archaeology
☒	☐	Animals and other organisms
☒	☐	Clinical data
☒	☐	Dual use research of concern
☒	☐	Plants

Methods

n/a	Involved in the study	
☒	☐	ChIP-seq
☒	☐	Flow cytometry
☒	☐	MRI-based neuroimaging

Antibodies

Antibodies used	anti-GAPDH (Cat# is HRP-60004, Proteintech Group Inc.); anti-hMEX3B (Cat# is WL3452579, Invitrogen), anti-HLA-A (Cat# is DF6890, Affinity). Horseradish peroxidase-conjugated anti-rabbit (Cat# is STAR121, Bio-Rad Laboratories Inc.) and anti-mouse (Cat# is AAC10P, Bio-Rad Laboratories Inc.)

Validation	validation statements on the manudacturer's websites.

Eukaryotic cell lines

Policy information about cell lines and Sex and Gender in Research

Cell line source(s)	HEK293T cell (lab maintained)
Authentication	None of the cell lines used were authenticated.
Mycoplasma contamination	mycoplasma contamination negative confirmed.
Commonly misidentified lines (See ICLAC register)	*Name any commonly misidentified cell lines used in the study and provide a rationale for their use.*

Plants

Seed stocks	This study doesn't include plants data
Novel plant genotypes	This study doesn't include plants data
Authentication	This study doesn't include plants data
